# Matrix metalloproteinase-9 (MMP-9) and tissue inhibitor of metalloproteinases 1 (TIMP-1) are localized in the nucleus of retinal Müller glial cells and modulated by cytokines and oxidative stress

**DOI:** 10.1371/journal.pone.0253915

**Published:** 2021-07-16

**Authors:** Eun-Jin Lee, Mengmei Zheng, Cheryl Mae Craft, Shinwu Jeong

**Affiliations:** 1 Mary D. Allen Vision Research Laboratory, USC Roski Eye Institute, Department of Ophthalmology, Keck School of Medicine of the University of Southern California, Los Angeles, CA, United States of America; 2 Department of Ophthalmology, Stanford University, Palo Alto, CA, United States of America; 3 Department of Integrative Anatomical Sciences, Keck School of Medicine of the University of Southern California, Los Angeles, CA, United States of America; University of Massachusetts Medical School, UNITED STATES

## Abstract

Matrix metalloproteinases (MMPs) are involved in the pathology of numerous inflammatory retinal degenerations, including retinitis pigmentosa (RP). Our previous work revealed that intravitreal injections with tissue inhibitor of metalloproteinases 1 (TIMP-1) reduce the progression of rod cell death and inhibit cone cell remodeling that involves reactive gliosis in retinal Müller glial cells (MGCs) in rodent models. The underlying cellular and molecular mechanisms of how TIMP-1 functions in the retina remain to be resolved; however, MGCs are involved in structural homeostasis, neuronal cell survival and death. In the present study, MMP-9 and TIMP-1 expression patterns were investigated in a human MGC line (MIO-M1) under inflammatory cytokine (IL-1β and TNF-α) and oxidative stress (H_2_O_2_) conditions. First, both IL-1β and TNF-α, but not H_2_O_2,_ have a mild *in vitro* pro-survival effect on MIO-M1 cells. Treatment with either cytokine results in the imbalanced secretion of MMP-9 and TIMP-1. H_2_O_2_ treatment has little effect on their secretion. The investigation of their intracellular expression led to interesting observations. MMP-9 and TIMP-1 are both expressed, not only in the cytoplasm, but also inside the nucleus. None of the treatments alters the MMP-9 intracellular distribution pattern. In contrast to MMP-9, TIMP-1 is detected as speckles. Intracellular TIMP-1 aggregation forms in the cytoplasmic area with IL-1β treatment. With H_2_O_2_ treatments, the cell morphology changes from cobbles to spindle shapes and the nuclei become larger with increases in TIMP-1 speckles in an H_2_O_2_ dose-dependent manner. Two TIMP-1 cell surface receptors, low density lipoprotein receptor-related protein-1 (LRP-1) and cluster of differentiation 82 (CD82), are expressed within the nucleus of MIO-M1 cells. Overall, these observations suggest that intracellular TIMP-1 is a target of proinflammatory and oxidative insults in the MGCs. Given the importance of the roles for MGCs in the retina, the functional implication of nuclear TIMP-1 and MMP-9 in MGCs is discussed.

## Introduction

Inherited forms of retinitis pigmentosa (RP) are initiated with rod cell death, followed by the rearrangement and degeneration of cone photoreceptor cells [1, 2]. RP is associated with inflammatory neuronal diseases involving increased proinflammatory cytokines and oxidative stress [3, 4]. The integrity and function of retinal neurons in RP are determined, not only by an internal balance between survival and death signaling, but also by a dynamic maintenance of retinal architecture composed of multiple neuronal cell types and surrounding extracellular matrix (ECM).

Retinal Müller glial cells (MGCs) are the most abundant resident macroglial cells and are closely associated with all neuronal cell types in the retina, providing vital functional roles in the healthy retina [5, 6]. In retinal degeneration, MGCs undergo reactive gliosis, a homeostasis-maintaining process to keep the functional integrity of the retina. MGCs are one of primary responders to the onset of rod cell death in RP retina [7]. However, too much gliosis can result in pathological effects [8]. Matrix metalloproteinase 9 (MMP-9) and tissue inhibitor of metalloproteinases 1 (TIMP-1) are expressed in MGCs [9, 10]. TIMP-1 cytokine-like activities can be exerted dependently or independently of the interaction with MMP-9 [11]. It still remains unknown how the expression and interaction of MMP-9 and TIMP-1 are regulated and affect the functions of MGCs in the retina. MGCs span the neuronal layers in the retina, maintaining functional and structural homeostasis. A spontaneously immortalized human MGC line (MIO-M1) retain the characteristics of primary isolated cells and express mature MG cell markers, including glutamine synthetase (GS), cellular retinaldehyde-binding protein (CRALBP), vimentin and epidermal growth factor receptor-(EGF-R) [12]. MMP-9 is rarely detected in healthy retina [13]; however, the upregulation of MMP-9 expression is associated with the retinal degeneration in rodent RP models [14–16].

Our previously published work in *rd*1 mouse and S334ter-line-3 transgenic rat retina provides convincing evidence that MMP-9 inhibitors have both a positive neuronal cell survival effect and a therapeutic potential to slow rod cell death, which is the initial hallmark of retinal degeneration. Using either a recombinant TIMP-1, a synthetic chemical compound SB-3CT, or clusterin [14, 15, 17], our observations documented that these compounds inhibit the cone mosaic rearrangements, an event observed prior to cone cell death in RP retina, by affecting the reactive gliosis of the MGCs in the retina [18, 19]. These cumulative results strongly implicate these reagents actively play a role in the MGCs response to RP progression by inhibiting MMP-9 activity.

Not only does TIMP-1 inhibit MMP-9 activities, but it has pleiotropic cytokine-like signaling activity that plays a critical role in the cellular metabolic status, including cell growth, apoptosis, differentiation, and angiogenesis [11]. These multi-functional roles are exhibited predominantly outside cells by modulating MMP protease activities and/or interacting with numerous, distinct cell surface signaling proteins [20]; however, TIMP-1 was also detected even within the nucleus in a few studies [21, 22].

In the present study, we hypothesized that MGCs express endogenous MMP-9 and TIMP-1, the expression of which is altered in retinal remodeling during retinal degeneration. We investigated how these proteins are expressed in human MGCs under normal, inflammatory, and oxidative stress conditions. We discovered that MMP-9 and TIMP-1 are localized inside the nucleus and secreted. Furthermore, we confirmed proinflammatory and oxidative stress modulates MMP-9 and TIMP-1 secretion levels and the TIMP-1’s intracellular distribution pattern.

## Materials and methods

### Reagents and chemicals

Dulbecco’s modified Eagle medium (DMEM) was obtained from Thermo Fisher Sci (Grand Island, NY). Fetal bovine serum (FBS) was from Atlanta Biologicals (Atlanta, GA). Human interleukin (IL)-1β, tumor necrosis factor (TNF)-α, pro-MMP-9 and pro-MMP-2 were purchased from R&D Systems (Minneapolis, MN). Chemical compounds, MTT (3-(4,5-dimethyl-2-thiazolyl)-2,5-diphenyl-2H-tetrazolium bromide), Sulforhodamine B (SRB), Hydrogen peroxide (H_2_O_2_), and Coomassie brilliant blue were purchased from Sigma-Aldrich Corp (St. Louis, MO).

### Cell culture

Human Müller cell line MIO-M1 (Moorfields Eye Hospital/Institute of Ophthalmology-Müller1) was provided by Dr. Hossein Ameri (University of Southern California) who purchased the cells from XIP (London, UK). The cells were maintained in regular Dulbecco’s modified Eagle’s medium (DMEM) containing 10% fetal bovine serum (FBS), 100 U/mL penicillin, and 100μg/mL streptomycin in a cell culture incubator at 37°C and 5% CO_2_. MIO-M1 cells were grown in 6-well plates (4x10^5^ cells/well), 8-well chamber slide (3x10^4^ cells/well), or 96-well plates (1x10^4^ cells/well) in regular or FBS-free media for 24 h before treating the cells with cytokines or H_2_O_2._

### Cell viability assay

The colorimetric MTT and SRB assays measure and assess cell metabolic activity, cytotoxicity or loss of viable cells. Briefly, MIO-M1 cells were grown in 96-well plates (1x10^4^ cells/well) for 24 h and then incubated with test reagents for 24 h in 10% FBS-containing or FBS-free media. After collecting conditioned media (CM) at 24 h post treatments of cytokine or H_2_O_2_, MTT solution was added into culture wells at a final concentration of 0.5mg/mL in media, and then incubated for 1 h. The purple formazan salt formed inside the cells was solubilized with DMSO and the color absorbance was read at the wavelength of 570nm using a plate reader, SoftMax Pro 7.1 (Molecular Devices, San Jose, CA). The background readings were subtracted from those of the test wells. Subsequently, the relative viable cell densities were obtained by comparing with those of untreated control cells. For SRB assay, the procedure is described in detail by Vinicha and Kirtikala [23]. Briefly, the cells in 96-well plates after treatments were fixed in 3.3% (w/v) trichloroacetic acid (TCA) solution for 1 h at 4°C, followed by staining with 0.057% (w/v) SRB solution for 30 min at room temperature (RT), and washing with 1% (w/v) acetic acid. The SRB bound to cellular proteins was dissolved in 10mM Tris base (pH 10.5) to read the color densities at 510nm. The data were treated as in MTT assay.

### Gelatin zymography

Gelatin zymography was utilized to detect the secretion of MMP-2 and MMP-9 according to the procedure described by Jeong *et al*. [24]. Zymography is widely used as a semi-quantitative in-gel assay to measure the MMP-2 and/or MMP-9 gelatinolytic activity. Due to the cleavage of gelatins, the MMP position and activity are revealed as a reduced stained band by Coomassie blue staining and the density is measured by densitometry. Briefly, MIO-M1 cells (1x10^4^ cells/well) were seeded onto 96-well plates and cultured for 24 h, and then washed twice with FBS-free media before adding cytokines or H_2_O_2._ The conditioned media (CM) were collected 24 h after the treatment. The CM of equal volumes were applied onto an 8% SDS/PAGE containing 0.1% gelatin (Sigma-Aldrich, St. Louis, MO). After electrophoresis under non-reducing conditions, the gels were washed in 2.5% Triton-X100 for 1 h at RT. The gels were then washed briefly with water, and then incubated overnight in the incubation buffer (10mM CaCl_2_, 50mM Tris-HCl [pH 7.5]) at 37°C, followed by Coomassie blue staining. The images of MMP bands on the gels were obtained using ChemiDocXRS+System (BioRad, Hercules, CA) and the densities of MMP bands were measured using Image J software version 1.53c (NIH, Bethesda, MD), followed by normalization by cell densities of individual samples. The relative densities of MMP bands were obtained by comparing with those of untreated control cells.

### Enzyme-Linked Immunosorbent Assay (ELISA)

The levels of TIMP-1 in the CMs were quantified using Human TIMP-1 ELISA kit (Ray Biotech, Peachtree Corners, GA) according to the protocol provided by the manufacturer. Briefly, the CM of equal volume were diluted by the sample dilution buffer provided, and applied to TIMP-1 antibody-coated 96-well plates at RT for 2.5 h. Subsequently, incubations with biotinylated TIMP-1 antibody for 1 h, and then HRP-streptavidin incubation for 45 min, were performed. Compound 3,3,5,5’-tetramethylbenzidine (TMB) was used to develop the luminescence to be detected at 450nm, using a plate reader, SoftMax Pro 7.1 (Molecular Devices, San Jose, CA). The final TIMP-1 concentrations were normalized by the cell densities of individual samples. The relative levels of TIMP-1 in the samples were obtained by comparing with those of untreated control cells.

### Immunohistochemistry

For immunohistochemistry (IHC), MIO-M1 cells (3x10^4^ cells/well) were seeded onto the 8-well chamber slides (BD Bioscience, Bedford, MA) 1 day before staining. Cells were washed in PBS, fixed with PBS containing 4% paraformaldehyde (PFA) at RT for 5 min, and followed by PBS buffer wash and incubation with methanol in -20^˚^C for 5 min. After the samples were incubated in the buffer containing 10% normal donkey serum (NDS) and 0.2% Triton X-100 in PBS for 30 min at RT, they were incubated in the same buffer at 4˚C overnight, with primary antibodies specific to MMP-2 (Millipore, dilution 1:500), MMP-9 (Abcam, 1:500), TIMP-1 (Millipore, 1:500), β-actin (Abcam, 1:1000), LRP-1 (Abcam, 1:500), CD63 (Millipore, 1:1000), and CD82 (Abcam,1:500) (S1 Table). Secondary antibodies, such as AlexaFluor488-conjugated or AexaFluor568-conjugated donkey or goat antibodies against rabbit or mouse IgG (Invitrogen, 1:500), were incubated for 2h at RT (S1 Table). The final slides were covered with Vectashield mounting medium with DAPI (Vector Labs, Burlingame, CA). The confocal microscopic images of slides were acquired using the Zeiss LSM-PC software under a Zeiss LSM 710 confocal microscope at 1μm Z-stacking intervals (Zeiss, San Diego, CA). The same image-acquiring conditions were used equally across the slide. Z-stacking microscopy was performed at 1μm intervals. The final images were processed using ZEN blue edition software (Zeiss, NY).

### Reverse transcription-PCR (RT-PCR)

Total RNA was prepared using TRI Reagent (Sigma Aldrich, St. Lois, MO) and High-Capacity RNA-to-cDNA™ Kit (ThermoFisher Sci., Chino, CA) were used for cDNA synthesis. RT-PCR performed using 10 ng RNA-equivalent cDNA with gene transcript-specific oligo DNA primers, using 2XPCR Master Mix (ThermoFisher Sci) to detect the mRNA transcripts of *LRP-1*, *CD63*, and *CD82*. The nucleotide sequences of the primers are described in S2 Table.

### Statistical analysis

One-way analysis of variance (ANOVA) and Fisher’s least significant difference procedure (LSD test) were performed to examine the differences among the test groups, using GraphPad Prism6 (La Jolla, CA, USA). The statistical values are presented as mean ± standard error (SE). Difference between the means of separate treatment groups was considered statistically significant at p-value (p) <0.05.

## Results

### Cytokines increase the secretion of MMP-9 and TIMP-1 from MIO-M1 cells

MGCs express MMP-9 and TIMP-1 *in vitro* [9, 10]. In the present study using MIO-M1 culture system, we examined if MGCs express MMP-9 and TIMP-1 in response to proinflammatory conditions such as exposure to cytokines and oxidative stress that precipitates retinal degeneration.

**Fig 1:**
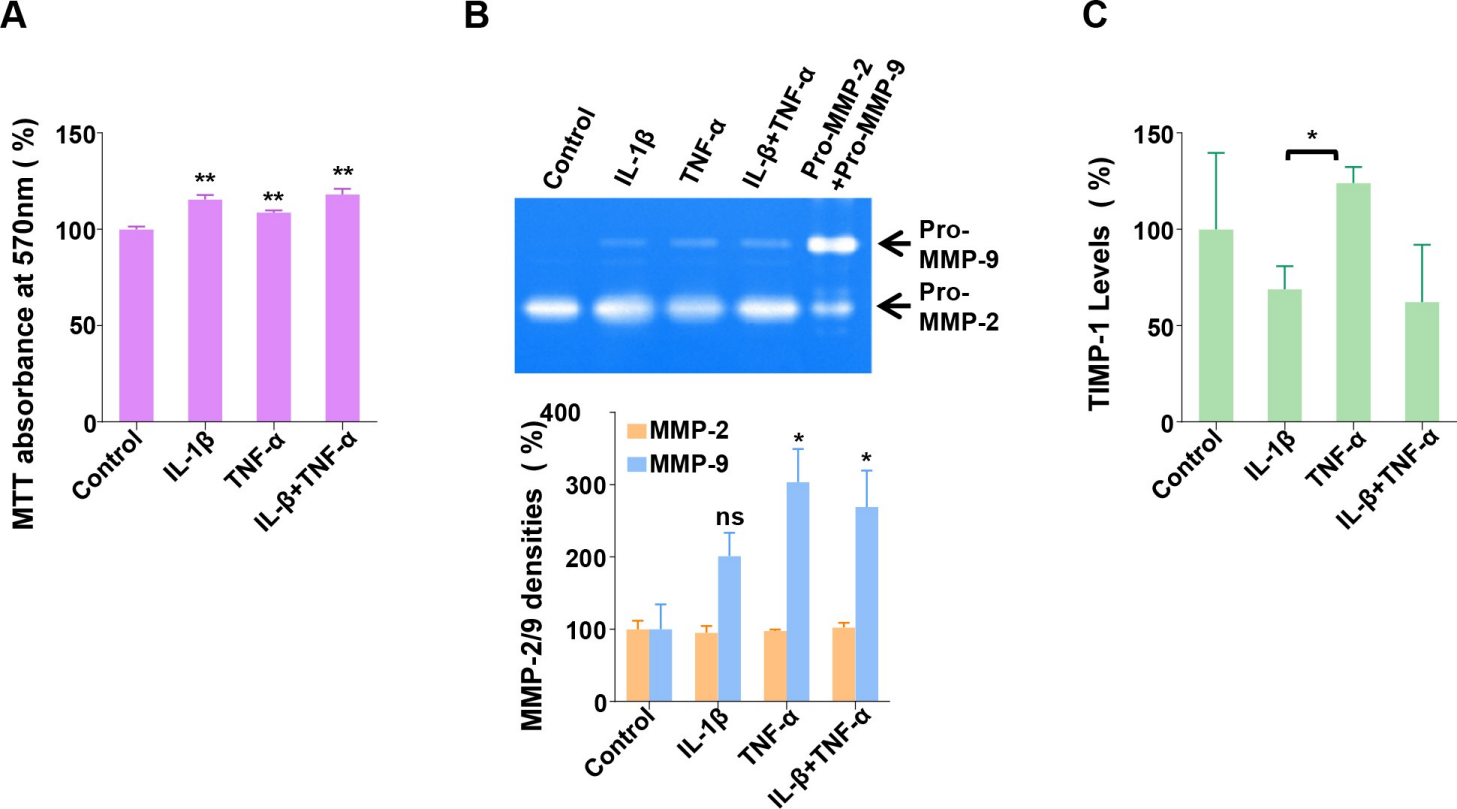
Cytokine regulation of the secretion of MMP-9 and TIMP-1 from MIO-M1 cells. (A) MIO-M1 cells were treated or untreated with IL-1β and TNF-α, at 10 ng/mL, alone or in combination, for 24 h, and then subjected to MTT assay to measure viable cell densities. Control refers to untreated samples. Relative cell densities are presented as % mean ± SE (n = 3), with Control set as 100%. **, p<0.01 (vs. Control) (B) The cells were treated in the same way as in (A), and then CMs were collected, to perform gelatin zymography assay. (Top) CMs of the same volume from each treatment was subjected to gelatin zymography. One representative zymogram is shown to visualize secreted MMPs. Recombinant human pro-MMP-2 and pro-MMP-9 (Pro-MMP-2+Pro-MMP-9) were applied to the gel before electrophoresis as a control. (Bottom) The density of each MMP band was quantified using Image J software. The individual values of MMP band densities were normalized by the corresponding MTT absorbance values (n = 3). ns, not significant (p>0.05 vs. Control); *, p<0.05 (vs. Control) (C) Secreted TIMP-1 levels in the CMs were measured using TIMP-1 ELISA assay (n = 3), and then normalized by the corresponding MTT absorbance values. *, p<0.05 (IL-1β vs. TNF-α).

When originally established, MIO-M1 cells were characterized by the expression of several Müller cell markers, including glutamine synthetase (GS), glial fibrillary acidic protein (GS), vimentin (VIM), α-smooth muscle actin (ACTA2), and epidermal growth factor receptor (EGFR) [12]. First, using RT-PCR technology, we verified the expression of these transcripts to authenticate the current cells (S1A Fig). Treating MIO-M1 cells with IL-1β or TNF-α, alone or in combination, at 10ng/mL in FBS-free media, for 24 h, we used the colorimetric MTT assay that measures cellular metabolic activities [25] and tested if the cytokine treatments affect the cell viability (Fig 1A). The two cytokine treatments increase the MTT absorbance: IL-1β increases it to 116% (p<0.01), compared with untreated control, and TNF-α, to 109% (p<0.01). The combination treatment also increases the growth rate by 118% (p<0.01), similarly by IL-1β alone. The results suggest that these cytokines increase the cell viability or proliferation of MIO-M1 cells. We utilized a second method to confirm these results, the SRB assay, which determines cell density based on cellular protein content measurement [23]. Cytokine-induced increases in cell densities were consistently observed (S1B Fig). To distinguish between proliferative or pro-survival effects, we measured cell density changes before and after IL-1β treatment at 0 h and 24 h. The results indicate that the cell density of untreated cells is reduced significantly over 24 h (p<0.05), which is preserved by IL-1β treatment (p<0.01) (S1C Fig). Altogether, the results from both assays suggest that these cytokines have an *in vitro* pro-survival effect on MIO-M1 cells in FBS-free media lacking growth factors. Next, using the CM collected from control and treated cell cultures, the secretion of MMP-2 and MMP-9 was examined using gelatin zymography. In Fig 1B (Top), secreted MMP-2 and MMP-9 are revealed on the gelatin zymogram. In the untreated control, only MMP-2 band is distinct with MMP-9 barely detectable. However, upon IL-1β or TNF-α treatment, MMP-9 protein bands become visible, indicating increased MMP-9 secretion by both cytokines. These secreted MMP-2 and MMP-9 co-migrate with purified recombinant pro-form of each protein, suggesting that they are secreted mainly in zymogens from MIO-M1 cells *in vitro*. In the lower panel of Fig 1B, the quantification of the densities of both MMP2 and MMP9 bands on zymograms was performed using Image J software. The results indicate that the secretion of MMP-2 is not affected by the cytokine treatments, and that the level of MMP-9 secretion is increased to 201% by IL-1β (p>0.05), and 304% by TNF-α (p<0.05), compared with untreated control. The combined treatment also increases the MMP-9 secretion to 270% (p<0.05). In Fig 1C, the levels of TIMP-1 in the CM were quantified by ELISA assay. To do this, the transcription and secretion of TIMP-1 were first confirmed by RT-PCR and immunoblot analysis using the CM, respectively (S1D Fig). The level of TIMP-1 secretion is reduced to 68% by IL-1β (not significant, p>0.05), compared with untreated control, whereas TNF-α increases the secretion to 124% (not significant, p>0.05). Even though the difference between the levels by both cytokines are significant (p<0.05), it is unclear if they have an opposite effect, as the reduction of TIMP-1 by the combined treatment is not significantly different from that by IL-1β treatment alone. These data demonstrate that both cytokines highly induce MMP-9 secretion, but they modulate the TIMP-1 secretions to a lesser extent by either increasing with TNF-α or decreasing with IL-1β.

### Endogenous MMP-9 and TIMP-1 are localized in the nucleus

**Fig 2:**
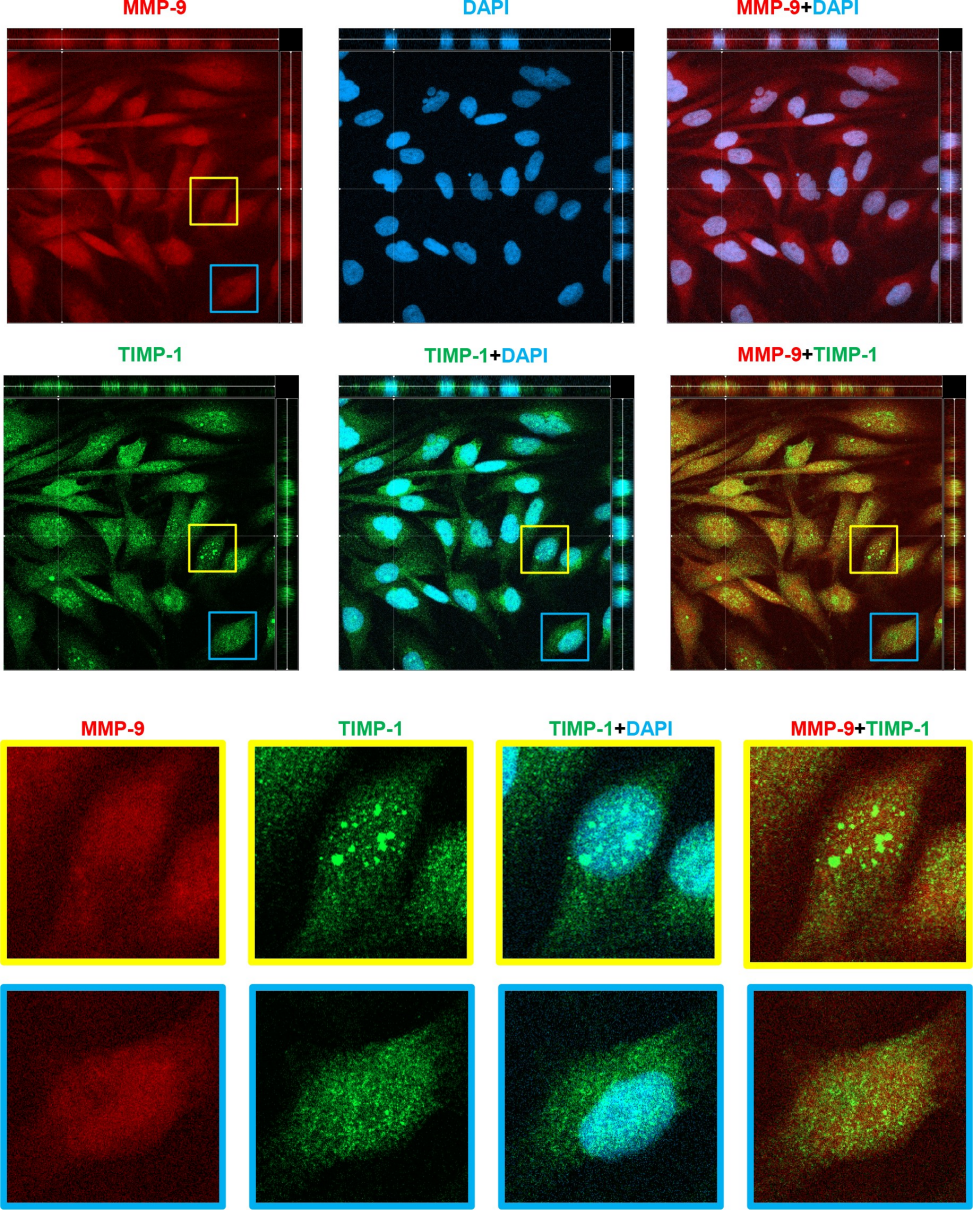
Intracellular compartmentalization of MMP-9 and TIMP-1 in MIO-M1 cells. MIO-M1 cells cultured in regular media were subjected to IHC fluorescent confocal microscopy with 1μm-interval Z-stacking. Primary antibodies specific to MMP-9 (red) and TIMP-1 (green) followed by the appropriate fluorescent secondary antibodies, as indicated in the methods, were used to dual-IHC staining of the cells. Nuclear region is defined by chromatin staining with DAPI (blue). Orthogonal projection of one focal plane is presented. Cells with two different patterns in nuclear TIMP-1 distribution are indicated by yellow and blue insets and enlarged in the bottom two rows. Scale bars, 50 μm.

Both MMP-9 and TIMP-1 have been detected inside cells in certain cell types, including neuronal cells, with functional implications [26]. We examined the intracellular expression pattern of these proteins in MIO-M1 cells grown in regular media by IHC with dual fluorescent immunological staining with MMP-9 and TIMP-1 antibodies. The nuclear region was identified by DAPI staining (Fig 2). The three-dimensional distribution of the proteins inside the cells with Z-stacking microscopy at 1 μm interval was performed. The orthogonal projections, reflecting 3-dimensional distribution, are presented. At the top of Fig 2, the general distribution of MMP-9 immunoreactivity (red) revealed on the horizontal plane is widely diffused covering cytoplasmic and nuclear area (blue), showing its presence beyond the nuclear area in MMP-9+DAPI superimposition. The same superimposition on the other two vertical planes shows nuclear magenta color throughout the vertical depth, suggesting that MMP-9 is present internally within the nuclei. TIMP-1 is also detected in both nuclear (TIMP-1+DAPI) and cytoplasmic areas. Dual localization of MMP-9 and TIMP-1 (MMP-9+TIMP-1) demonstrates that they are co-localized within the same cells. Interestingly, TIMP-1 appears in punctate or speckled patterns throughout cytoplasmic and nuclear area: the sizes of “TIMP-1 speckles” vary, so that some cells have bigger ones with various sizes, especially, inside the nuclei (yellow inset) and the other, not (blue inset). Those speckles are not observed with the MMP-9 localization, suggesting that TIMP-1 may have a distinct role independently of its MMP-9 binding activity. MMP-2 was also examined and is also localized inside the nucleus (S2 Fig).

### Cytokine treatment induces intracellular TIMP-1 aggregation

**Fig 3:**
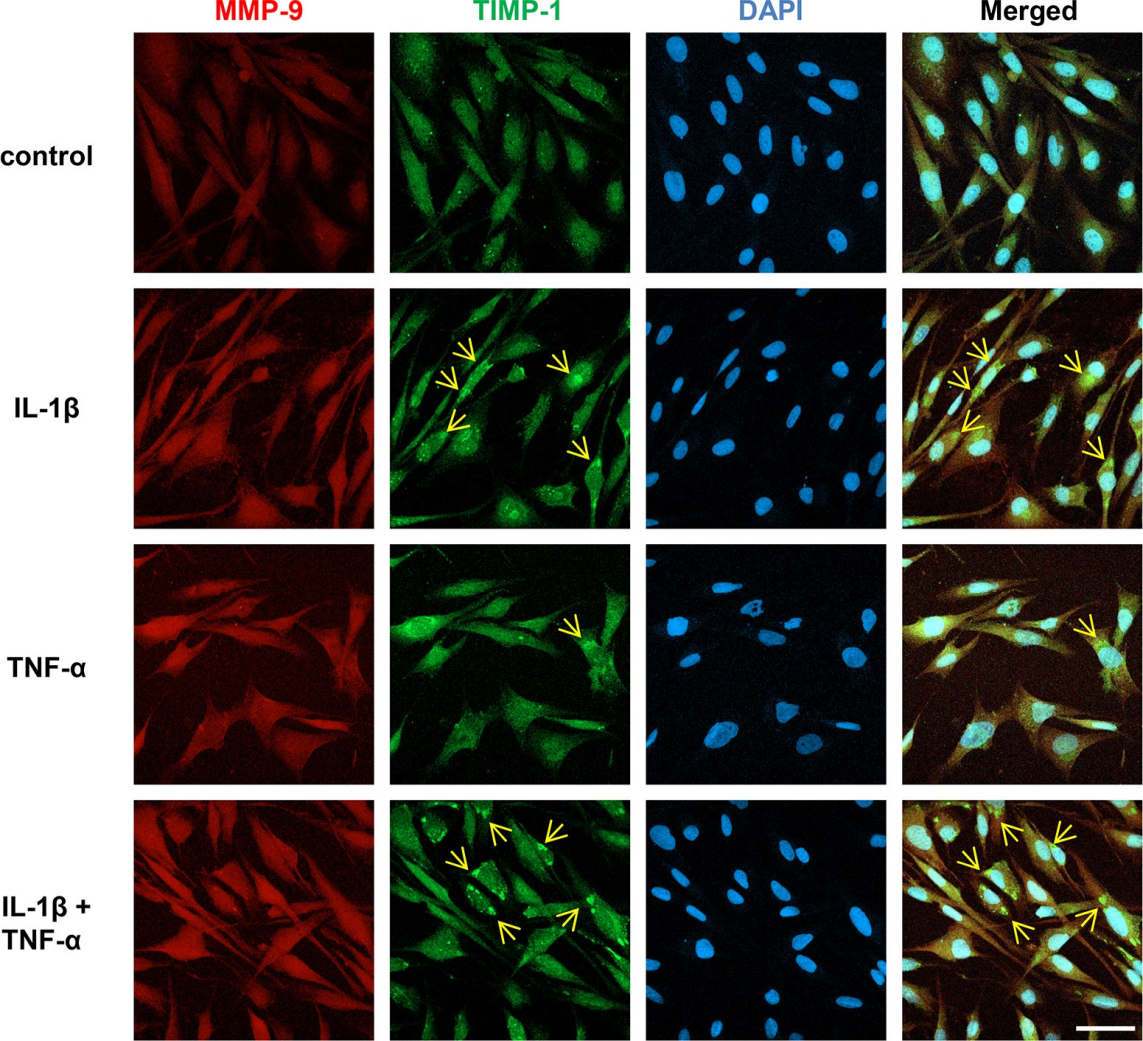
Effect of cytokine treatment on the intracellular MMP-9 and TIMP-1 distribution. MIO-M1 cells were treated or untreated with IL-1β and/or TNF-α, each at 10 ng/mL in FBS-free media, alone or in combination, for 24 h, and then IHC confocal microscopy was performed to detect intracellular MMP-9 (red) and TIMP-1 (green) by dual-IHC staining using their specific primary and secondary fluorescent antibodies. Nuclear region is defined by chromatin staining with DAPI (blue). Representative micrographs of each treatment group are presented. Arrows point to immunoreactiveTIMP-1 aggregates. Scale bars, 50 μm.

Next, we tested the effect of the cytokine treatment on the intracellular distribution of MMP-9 and TIMP-1. MGCs were treated with IL-1β and/or TNF-α, each at 10ng/mL, for 24 h before processing for IHC using MMP-9 and TIMP-1 antibodies. In Fig 3A, the expression of MMP-9 is similar in untreated control and cytokine-treated cells, being diffusely distributed in the nucleus and cytoplasm, indicating that the cytokine treatment has no apparent effect on the MMP-9 distribution within the cells. In untreated control, the expression patterns of TIMP-1 are similar to those of MMP-9. However, in contrast, in IL-1β-treated cells, “aggregated” TIMP-1-immunoreactivities (indicated by arrows) are detected in the cytoplasmic area in some cells, whereas these are fewer with TNF-α treatment. These aggregates are also prominent in the cells with combined treatment of IL-1β and TNF-α. Since there is no apparent MMP-9 aggregation in the same treatments, the results suggest that the TIMP-1 aggregation occurs independently of MMP-9. The intracellular MMP-2 distribution, like MMP-9, is not affected by cytokine treatment, compared with control (S3 Fig).

### Oxidative stress induces cell morphological changes and formation of TIMP-1 nuclear speckles

**Fig 4:**
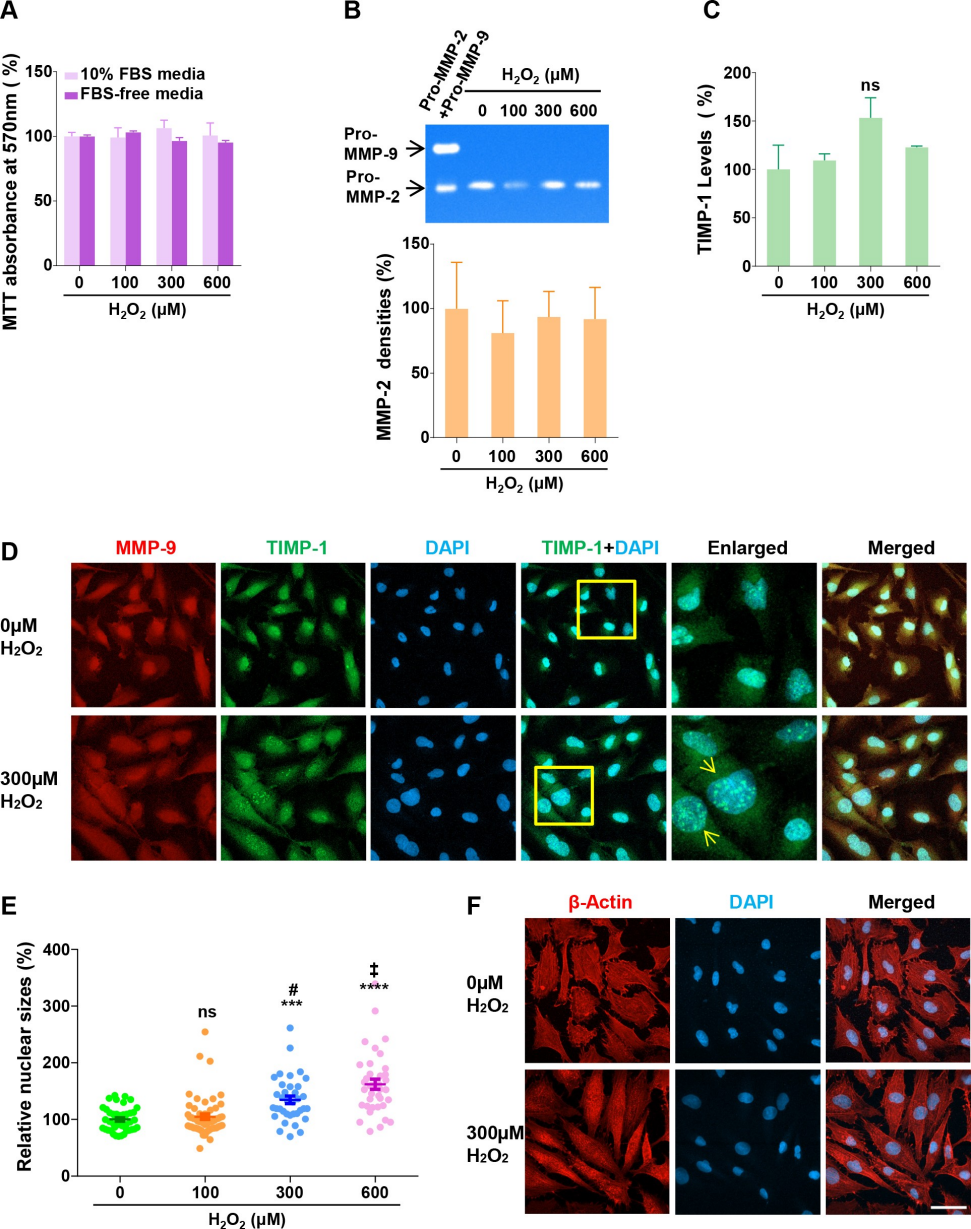
Effect of oxidative stress on intracellular MMP-9 and TIMP-1. (A) MIO-M1 cells were cultured in the presence of H_2_O_2_ at indicated concentrations in the regular (10% FBS) or FBS-free media, for 24 h, and then subjected to MTT assay to measure viable cell densities. Relative cell densities are presented as % mean ± SE (n = 3), with control (0μM) set as 100%. (B) The cells were treated in the same way as in (A), and then CMs were collected, to perform gelatin zymography and ELISA assay. (Top) the CMs of the same volume from each treatment was subjected to gelatin zymography. One representative zymogram is shown to visualize secreted MMPs. A mixture of recombinant human pro-MMP-2 and pro-MMP-9 (Pro-MMP-2+Pro-MMP-9) were applied to the gel prior to electrophoresis as a MMPs marker control. (Bottom) The density of each MMP band was quantified using Image J software (n = 3). The individual values of MMP band densities were normalized by the corresponding MTT absorbance values. (C) Secreted TIMP-1 levels in the same CMs were measured using TIMP-1 ELISA assay (n = 3). The individual ELISA values were normalized by the corresponding MTT absorbance values. ns, not significant (p>0.05, vs. 0μM). (D) MIO-M1 cells were cultured in the presence of 300 μM H_2_O_2_ for 24 h, and then subjected to IHC confocal microscopy, for dual IHC staining with MMP-9 (red) and TIMP-1 (red) antibodies. Insets in TIMP-1+DAPI micrograms are enlarged, and arrows point to the bigger nuclei with increased TIMP-1 speckles. (E) Nuclear sizes in the micrograms of the cells treated with increasing concentrations of H_2_O_2_ were measured, using Image J software. Relative nuclear sizes are presented as % mean ± SE. ns, not significant (p>0.05 vs. 0μM); ***, p<0.001 (vs. vs. 0μM; ****, p<0.0001 (vs. 0μM); #, p<0.001 (vs. 100μM); ‡, p<0.01 (vs. 300μM). The numbers of randomly selected nuclei measured were 43 (0μM), 55 (100μM), 36 (300μM), and 37 (600μM). (F) β-actin (red) antibody was used to perform a confocal microscopy after 300μM H_2_O_2_ treatment for 24h. Representative micrographs of untreated (0μM) and treated (300μM) groups are presented. bars, 50 μm.

Oxidative stress is also one of the key causative factors in many forms of retinal degeneration [27]. We first tested the oxidative stress effect on the MIO-M1 viability by MTT assay, after treating H_2_O_2,_ at 0μM, 100μM, 300μM, and 600μM, in 10% FBS-containing regular culture media or FBS-free media for 24 h (Fig 4A). The results from the assay demonstrate that H_2_O_2_ treatment has little effect on the cell viability in both culture conditions. This conclusion was also confirmed using SRB assay (S4A Fig). The cells were grown in FBS-free media to measure the secretions of the MMP proteins, because the FBS contains bovine MMP-2 and MMP-9, which obscure those from MIO-M1 cells on zymogram (S4B Fig). In Fig 4B, gelatin zymography only reveals MMP-2 bands, whose densities were measured to show that the levels of MMP-2 secretion are not affected. After H_2_O_2_ treatment, the secreted TIMP-1 was measured using an ELISA assay (Fig 4C), indicating that the TIMP-1 secretion is increased by 9% (0μM), 53% (300μM), 23% (600μM), with no statistical significance. Therefore, in contrast to cytokine treatments above, H_2_O_2_ treatment does not influence the secretions of either MMP-9 or TIMP-1.

Next, we performed IHC using antibodies against MMP-9 and TIMP-1 in cells treated with H_2_O_2_ to investigate the expression of MMP-9 and TIMP-1. In Fig 4D, the representative micrograms from the 0μM and 300μM-treated cells are presented. No detectable changes are seen between treated and untreated cells in the expression pattern of MMP-9 or TIMP-1. Interestingly, however, some cells in the treated samples contain bigger nuclei with increased TIMP-1 speckle numbers (indicated by arrows in enlarged micrograms). Representative images of the nuclei at different concentrations of H_2_O_2_ are presented in S4C Fig, and the nuclear sizes in treatment groups were measured using Image J software (Fig 4E). The sizes are increased by 5% (not significant, p>0.05), 35% (p<0.001), and 62% (p<0.0001), by 100μM, 300μM, and 600μM treatments, respectively, compared with untreated control. The nuclear size increase shows a dose-dependency, with p<0.001 (100μM vs. 300μM) and p<0.01 (300μM vs. 600μM). These results suggest that oxidative stress leads to the formation and accumulation of nuclear TIMP-1 speckles in MIO-M1 cells. Next, we tested if the oxidative stress condition affects cellular morphologies with IHC with an antibody against β-actin, a major component of cell cytoskeleton (Fig 4F). Untreated cells appear with cobble-shape morphologies, whereas the treated ones develop spindle shapes, suggesting H_2_O_2_ induces cell morphological changes.

### TIMP-1 cell surface receptor proteins are expressed in MIO-M1 cells

**Fig 5:**
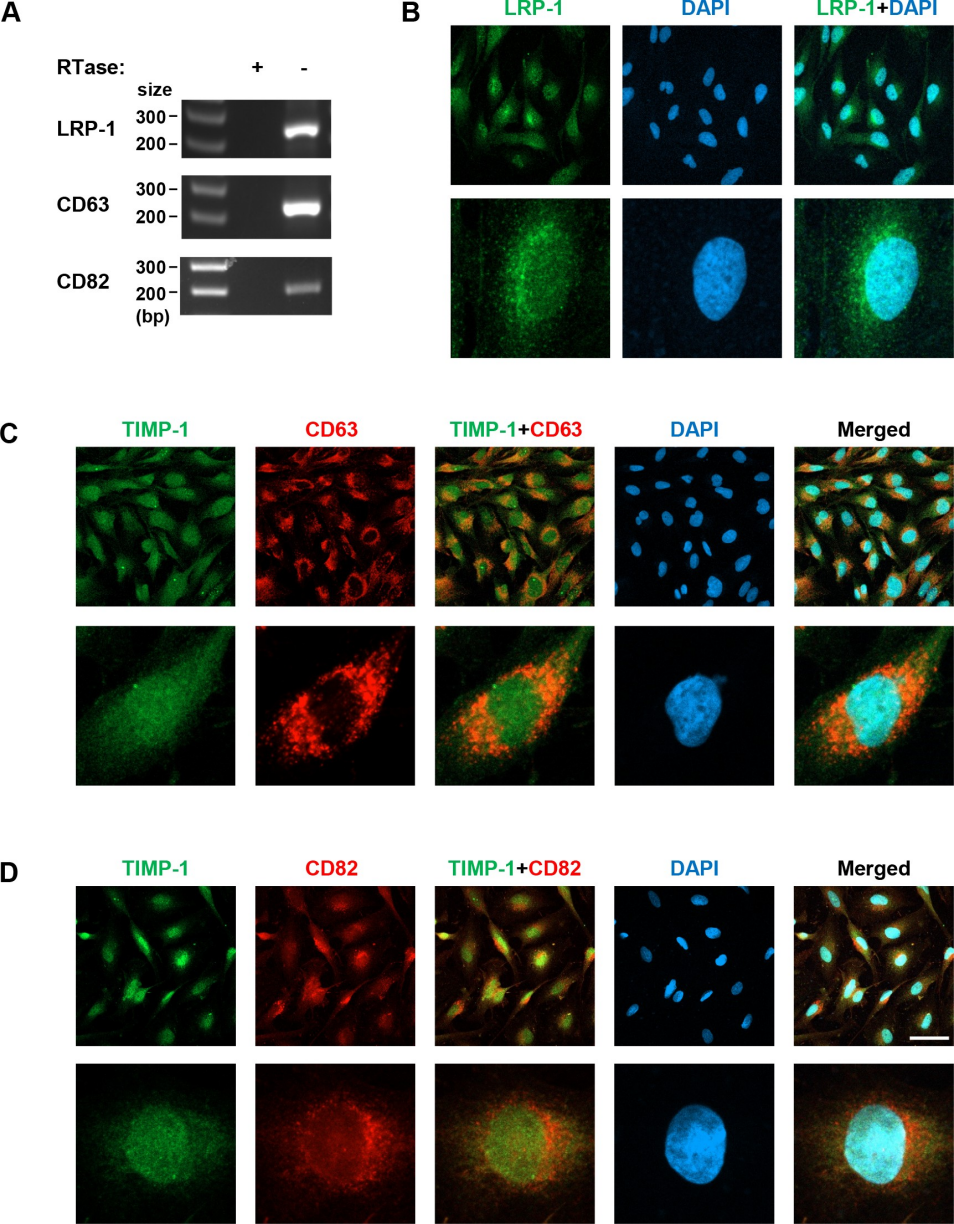
TIMP-1 cell surface receptors in MIO-M1 cells. (A) Total RNAs were isolated from MIO-M1 cells and their cDNAs were used for RT-PCR with gene transcript-specific PCR primers. The cDNA samples synthesized without reverse transcriptase (-RTase) were included as negative controls. Amplified cDNA products were resolved in agarose gel, with the DNA size markers (size). bp, base pairs. The cells grown in the regular media were subjected to IHC to detect LRP-1 (green) (B), CD63 (red) (C), and CD82 (red) (D), and the latter two were dual-IHC stained with TIMP-1 (green) antibody. Scale bars, 50 μm.

MMPs and TIMPs enter the cells through cell surface receptor-mediated endocytic processes [28, 29]. The receptors such as LRP-1 (CD91) [29], CD63 [30], and CD82 [31] are known to mediate TIMP-1 endocytic processes. We examined if these receptors are also present in the nuclei of MIO-M1. Using RT-PCR with gene transcript-specific DNA oligo primers, we first tested if these receptor proteins are expressed in MIO-M1 cells. In Fig 5A, the RT-PCR results reveal the expression of all three receptor transcripts at the transcriptional level. Then, we performed IHC with antibodies specific to these proteins. In Fig 5B, the MIO-M1 cells were immunologically stained with LRP-1 Ab. The fluorescent immunoreactivity of LRP-1 is detected with DAPI within the nuclei as well as in the cytoplasm. Both TIMP-1 and LRP-1 antibodies used in the current study were rabbit polyclonal, so that co-immunohistochemical staining was not feasible. In Fig 5C, the expression pattern of CD63 and TIMP-1 were probed at the same time with co-immunohistochemical staining. Results indicate that CD63 is located predominantly in the cytoplasmic area with weak punctate staining in the nuclei. Dual localization image of CD63 and TIMP-1 proteins indicates they are highly dual localized in the cytoplasm, suggesting they are interacting inside the cells. In Fig 5D, the expression pattern of CD82 looks similar to that of LRP-1 expression, revealing its nuclear and, more predominantly, perinuclear localizations.

## Discussion

Our extensive work in rodent models of RP clearly validated that intravitreal injections of recombinant TIMP-1 protein and other MMP-9 inhibitors slow down rod photoreceptor cell death [12–14]. Subsequently, TIMP-1 affects cone cell mosaic remodeling and second-order neuronal structures, which involves a gliotic response in MGCs [13, 15, 16]. However, unanswered questions linger of how MMP-9 and TIMP-1 are endogenously expressed and interact in healthy and degenerating retina. In fact, sustained chronic inflammation is a clinical observation in the retinas of RP patients [4]. Also, oxidative stress has been implicated in retinal diseases, including RP and age-related macular degeneration (AMD) [3, 32].

To obtain insights into these unresolved cellular pathways, we used a well-established *in vitro* MGC culture system to study the regulation and interaction of MMP-9 and TIMP-1 under normal, inflammatory, and oxidative conditions. The magnitude of the MMP-9 and TIMP-1 secretion is different in MGCs in response to cytokine treatments (Fig 1). The secretion of MMP-9 is increased 2 to 3-fold with IL-1β or TNF-α treatment; however, TIMP-1 secretion is increased just slightly with TNF-α or rather decreased with IL-1β, suggesting that MMP-9 and TIMP-1 are secreted disproportionately from the cells upon cytokine treatments. Our results may explain, at least partially, the previous observation that the retinal protein extracts from *rd*1 mouse have an imbalanced level of MMP-9 over TIMP-1, compared with those from WT mouse [24]. Another interesting observation of IL-1β-induced cytoplasmic TIMP-1 aggregation (Fig 3) may also provide a mechanism by which this imbalanced secretion occurs, as TIMP-1 aggregates may fail to be secreted, resulting in the reduction of TIMP-1 secretion. Such aggregation may also reduce TIMP-1 abundance to bind to and inhibit MMP-9, so that MMP-9 may become activated easily inside and outside of the MG cells. In support of this idea, aggregated TIMP-1 does not inhibit MMP-9 activity *in vitro* [33]. Physiological balance of MMPs and TIMPs is critical for the homeostasis in tissue structures. Considering the importance of the MGCs for the retinal structure maintenance, we propose that disproportionate MMP-9 secretion from these cells may contribute to the retinal architectural alteration in inflammatory RP conditions. We also envision that cytokine-induced aggregation of TIMP-1 in MGCs may be a novel mechanism involved in the inflammatory process in retinal degeneration, which may be associated with endoplasmic reticulum (ER) stress [34]. Further investigation of intracellular MMP-9 activation accompanying TIMP-1 aggregation is warranted.

Coupled or uncoupled to its inhibitory activity of MMP-9, TIMP-1 exerts cytokine-like pleiotropic functions, being involved in cell growth, differentiation, and apoptosis [11, 35]. Translocation and activation of microglial cells are observed in rodent RP retina [36], and activated microglial cells secrete proinflammatory cytokines, including TNF-α and IL-1β [37–39]. Light-induced photoreceptor death study using Zebrafish has demonstrated that dying photoreceptor cells produce TNF-α, which in turn induces its own expression from MGCs [40].

Our current work demonstrates, for the first time, that TIMP-1 and MMP-9 are co-localized in cell nuclei (Fig 2). There are twenty four MMP subtypes in human, typically secreted or membrane-bound [41]. To date, however, nine MMP subtypes have been detected in the nucleus of certain cell types including neurons: MMP-1 [42], MMP-2 [43–46], MMP-3 [47], MMP-9 [43–45, 48], MMP-10 [42], MMP-12 [49], MMP-13 [50], MMP-14 (MT1-MMP) [51], and MMP-15 (MT2-MMP) [42]. Nuclear MMPs are suggested to participate in apoptosis, cell migration, gene expression, and genetic instability [43, 44, 47, 52–54]. MMP-9 is detected within the S-phase nuclei of differentiating myoblasts [48], and its nuclear accumulation interferes with oxidative DNA repair, leading to neuronal cell death [44], suggesting that it may be involved in the cell fate determination depending on physiological conditions. Mechanisms by which they are localized to the nucleus are poorly addressed, except for MMP-3 that has a functional nuclear localization signal sequence shown in Chinese hamster ovary cells [47]. MMP-9 endocytosis is mediated by the LRP-2 receptor [55]; however, it remains to be tested if this receptor is involved in MMP-9 nuclear translocation. TIMP-1 has specific cell surface receptors, including LRP-1 [29], CD63 [30], and CD82 [31], which mediate TIMP-1 endocytosis. The intracellular domain of LRP-1 is localized to the nucleus, repressing interferon-γ transcription [56]. Our data reveal these three receptors are expressed in MIO-M1 cells, and that LRP-1 and CD82 have nuclear localization properties (Fig 5). The nuclear co-localization of TIMP-1 and its receptor, CD82, in MG cells is an interesting observation. TIMP-1-CD82 interaction and cytoplasmic translocation from the cell surface was previously observed in human pancreatic cancer tissues [31], reinforcing our observation. Furthermore, secreted TIMP-1-EGFP hybrid protein was shown to translocate into the nucleus of a human breast cancer cell line MCF-7 [18]. Additional studies detected nuclear TIMP-1 in endothelial cells and neurons [46] and proliferating gingival fibroblasts [19, 57]. At present, we tentatively propose two mechanisms to explain how MMP-9 and TIMP-1, which has no conventional nuclear localization signal, enters into the nuclei of MG cells: it may be from the cytoplasmic membrane of the cells via endocytic receptor-mediated process, and/or by a retrograde nuclear import process through non-classical nuclear localization, for example, aided by importins [58].

The current experiments demonstrate a novel observation that TIMP-1 is diffused in punctate structures of heterogenous sizes throughout MIO-M1 cells (Fig 2). At present, we identify them as “speckles” and propose that TIMP-1 speckles resemble paraspeckles in nucleoplasm or stress granules in cytoplasm, which change in number and size depending on cell cycle phases and metabolic demands [59, 60]. Compromised photoreceptors generate reactive oxygen species (ROS) [61–63] and oxidative stress is a key factor in retinal degeneration onset and progression [3, 64]. When cells are challenged with H_2_O_2_, many biological pathways are activated as a survival mechanism [65, 66]. For instance, MGCs respond to increased oxidative stress by changing their gene expression profile associated with cell protection [67, 68]. H_2_O_2_-induced oxidative stress induces cell morphological changes to spindle shapes in MIO-M1 cells and increases the formation of TIMP-1 speckles in the nucleus (Fig 4). Current H_2_O_2_-induced morphological change was seen similarly in *in vitro* MG-mesenchymal transition by TGF-β1 treatment to MIO-M1 cells [69]. Human MCF-7 cancer cells were recently demonstrated to undergo epithelial-mesenchymal transition by H_2_O_2_ treatment [70]. It would be interesting, therefore, to test if oxidative stress is involved in MG gliotic changes. In other studies, nuclear speckles have been observed to contain accumulated DYRK1A protein kinases playing a role in transcript splicing [71], and RNA polymerases in *T*. *spiralis*-infected muscle cells, where the nuclei are also enlarged, reflecting the elevation of host cell transcription [72]. Based on our observations, we propose that TIMP-1 dynamically participates in speckle formation with other nuclear or cytoplasmic proteins to cope with metabolic demands and oxidative stress challenges. The nature and function of the TIMP-1 speckles in MG cells warrant further studies.

In summary, our ongoing experiments lead us to propose that intracellular TIMP-1 plays critical functions in sustaining MGCs’ cellular activities and adaptive response to oxidative stress, which parallels the genetic alterations and subsequent protein expression changes that occur within MGCs during retinal degeneration in rodent RP models [73]. Our *in vitro* cell culture studies reinforce that TIMP-1 cytokine-like activities are complex and exerted dependently or independently of the interaction with MMP-9 [11]. TIMP-1, as a strong inhibitor of MMP-9 activation and activity, keeps the intracellular and nuclear MMP-9 activation status under surveillance in a healthy retina. However, such a role is compromised due to its aggregation when MGCs are exposed to increased cytokines under inflammatory conditions. TIMPs have an oligonucleotide/ oligosaccharide-binding (OB) fold in the N-terminal part of the protein [35, 74, 75], whose roles in TIMP-1 function have not been explored. Many DNA-damage checkpoint and DNA repair proteins possess the OB fold domain, which is critical for protein-protein, -RNA, and -DNA interactions [76]. We hypothesize that the nuclear TIMP-1 speckles participate in nuclear chromatin activities such as gene expression and/or DNA damage repair, interacting with proteins/RNA/DNA through its OB fold domain. Oxidative stress can induce DNA damage and gene transcriptional changes associated with cell death or survival choices [77, 78]. Nuclear speckle formations are associated with the events of the DNA repair and gene transcription, particularly pre-mRNA splicing [59, 79]. The accumulation of nuclear TIMP-1 speckles may occur to pre-mRNA splicing sites of a subset of genes associated with gliotic response of MGCs to the oxidative stress. Future studies will address the roles of MGCs in healthy and degenerating retinas and provide potential therapeutic targets to delay retinal degeneration.

## Supporting information

S1 Fig Verification of MIO-M1 cells and cytokine treatment. (A) Total RNA was isolated from MIO-M1 cells to synthesize cDNA, which was used for transcript-specific RT-PCR for Müller glial cell marker proteins. Reverse transcriptase-omitted samples (-RTase) were included as a negative control. GS, glutamine sythetase; GFAP, glial fibrillary acidic protein; VIM, vimentin; ACTA2, α-smooth muscle actin; EGFR, epidermal growth factor receptor. (B) MIO-M1 cells were treated or untreated with IL-β and TNF-α, at 10 ng/mL, alone or in combination, for 24 h, and then subjected to SRB assay to measure viable cell densities. Control refers to untreated samples. Relative cell densities are presented as % mean ± SE (n = 3), with Control set as 100%. IL-1β increases the SRB absorbance to 129% (p<0.01 vs. Control), and TNF-α, to 111% (p>0.05 vs. Control). The combination treatment also increases the growth rate by 128% (p<0.01 vs. Control); **, p<0.01 (vs. Control). (C) MIO-M1 cells were cultured in FBS-free media for 0 h and 24 h with and without 10 ng/mL IL-1β treatment, followed by SRB assay (n = 6). The cell density was reduced to 94% (p<0.05) at 24h from 100% at 0h without IL-1β treatment; however, the density was preserved at 104% (p<0.01) at 24 h with the treatment. *, p<0.05; **, p<0.01; ns, not significant (p>0.05). (D) The expression of TIMP-1 from MIO-M1 cells was confirmed. (Left) transcript-specific RT-PCR using cDNA. (Right) Immunoblot analysis (WB) was done using the conditioned media with anti-TIMP-1 antibody (Cell Signaling Tech, Catalog number:8946), TIMP-1 protein band is indicated by an arrow.
(TIF)

S2 Fig Intracellular distribution of MMP-2 in MIO-M1 cells. MIO-M1 cells cultured in regular media were subjected to IHC fluorescent confocal microscopy. The cells were immunohistologically stained with MMP-2 antibody (red) to localize the proteins. Nuclear region is defined by chromatin staining with DAPI (blue). Scale bar, 50 μm.
(TIF)

S3 Fig Effect of cytokine treatment on the intracellular MMP-2 distribution. MIO-M1 cells were treated or untreated with IL-1β and/or TNF-α, each at 10 ng/mL in FBS-free media, alone or in combination, for 24 h, and then IHC was performed to detect intracellular MMP-2 (red). Nuclear region is defined by chromatin staining with DAPI (blue). Representative micrograms of each treatment group are presented. Scale bar, 50 μm.
(TIF)

S4 Fig Effect of oxidative stress on MIO-M1 proliferation and intracellular TIMP-1 expression. (A) MIO-M1 cells were treated with H_2_O_2_ at 0μM, 100μM, 300μM, and 600μM in FBS-free media, for 24 h, and then subjected to SRB assay to measure cell densities. Relative cell densities are presented as % mean ± SE (n = 3), with control (0μM) set as 100%. There are no significant changes among treatment groups in SRB absorbance value. (B) Standard culture media containing 10% FBS were subjected to gelatin zymography to show that bovine serum contains MMP-2, which has a molecular size similar to human MMP-2. (C) MIO-M1 cells were cultured in the presence of H_2_O_2,_ at 0μM and 100μM, in FBS-free media, for 24 h, and then subjected to IHC for TIMP-1 (green) and DAPI (blue) micrograms. Single cell micrograms are presented. Scale bar, 10 μm.
(TIF)

S1 Table List of primary and secondary antibodies. (DOCX)

S2 Table List of reverse transcription-PCR primers. (DOCX)

S1 Raw images (PDF)

## References

[1] Jones BW, Marc RE. Retinal remodeling during retinal degeneration. Experimental eye research. 2005;81(2):123–37. doi: 10.1016/j.exer.2005.03.006 15916760

[2] Marc RE, Jones BW. Retinal remodeling in inherited photoreceptor degenerations. Molecular neurobiology. 2003;28(2):139–47. doi: 10.1385/MN:28:2:139 14576452

[3] Donato L, Scimone C, Nicocia G, D’Angelo R, Sidoti A. Role of oxidative stress in Retinitis pigmentosa: new involved pathways by an RNA-Seq analysis. Cell Cycle. 2019;18(1):84–104. doi: 10.1080/15384101.2018.1558873 30569795 PMC6343708

[4] Yoshida N, Ikeda Y, Notomi S, Ishikawa K, Murakami Y, Hisatomi T, et al. Clinical evidence of sustained chronic inflammatory reaction in retinitis pigmentosa. Ophthalmology. 2013;120(1):100–5. doi: 10.1016/j.ophtha.2012.07.006 22986109

[5] Bringmann A, Pannicke T, Grosche J, Francke M, Wiedemann P, Skatchkov SN, et al. Muller cells in the healthy and diseased retina. Progress in retinal and eye research. 2006;25(4):397–424. doi: 10.1016/j.preteyeres.2006.05.003 16839797

[6] de Hoz R, Rojas B, Ramirez AI, Salazar JJ, Gallego BI, Trivino A, et al. Retinal Macroglial Responses in Health and Disease. Biomed Res Int. 2016;2016:2954721. doi: 10.1155/2016/2954721 27294114 PMC4887628

[7] Hippert C, Graca AB, Barber AC, West EL, Smith AJ, Ali RR, et al. Muller glia activation in response to inherited retinal degeneration is highly varied and disease-specific. PloS one. 2015;10(3):e0120415. doi: 10.1371/journal.pone.0120415 25793273 PMC4368159

[8] Bringmann A, Iandiev I, Pannicke T, Wurm A, Hollborn M, Wiedemann P, et al. Cellular signaling and factors involved in Muller cell gliosis: neuroprotective and detrimental effects. Progress in retinal and eye research. 2009;28(6):423–51. doi: 10.1016/j.preteyeres.2009.07.001 19660572

[9] Limb GA, Daniels JT, Pleass R, Charteris DG, Luthert PJ, Khaw PT. Differential expression of matrix metalloproteinases 2 and 9 by glial Muller cells: response to soluble and extracellular matrix-bound tumor necrosis factor-alpha. The American journal of pathology. 2002;160(5):1847–55. doi: 10.1016/s0002-9440(10)61131-5 12000736 PMC1850886

[10] Gerhardinger C, Costa MB, Coulombe MC, Toth I, Hoehn T, Grosu P. Expression of acute-phase response proteins in retinal Muller cells in diabetes. Invest Ophthalmol Vis Sci. 2005;46(1):349–57. doi: 10.1167/iovs.04-0860 15623795

[11] Ries C. Cytokine functions of TIMP-1. Cellular and molecular life sciences: CMLS. 2014;71(4):659–72. doi: 10.1007/s00018-013-1457-3 23982756 PMC11113289

[12] Limb GA, Salt TE, Munro PM, Moss SE, Khaw PT. In vitro characterization of a spontaneously immortalized human Muller cell line (MIO-M1). Invest Ophthalmol Vis Sci. 2002;43(3):864–9. 11867609

[13] De Groef L, Andries L, Lemmens K, Van Hove I, Moons L. Matrix metalloproteinases in the mouse retina: a comparative study of expression patterns and MMP antibodies. BMC ophthalmology. 2015;15:187. doi: 10.1186/s12886-015-0176-y 26714639 PMC4696081

[14] Kim HS, Vargas A, Eom YS, Li J, Yamamoto KL, Craft CM, et al. Tissue inhibitor of metalloproteinases 1 enhances rod survival in the rd1 mouse retina. PloS one. 2018;13(5):e0197322. doi: 10.1371/journal.pone.0197322 29742163 PMC5942829

[15] Shin JA, Kim HS, Vargas A, Yu WQ, Eom YS, Craft CM, et al. Inhibition of Matrix Metalloproteinase 9 Enhances Rod Survival in the S334ter-line3 Retinitis Pigmentosa Model. PloS one. 2016;11(11):e0167102. doi: 10.1371/journal.pone.0167102 27893855 PMC5125676

[16] Ahuja S, Ahuja P, Caffe AR, Ekstrom P, Abrahamson M, van Veen T. rd1 mouse retina shows imbalance in cellular distribution and levels of TIMP-1/MMP-9, TIMP-2/MMP-2 and sulfated glycosaminoglycans. Ophthalmic research. 2006;38(3):125–36. doi: 10.1159/000090533 16374054

[17] Vargas A, Kim HS, Baral E, Yu WQ, Craft CM, Lee EJ. Protective effect of clusterin on rod photoreceptor in rat model of retinitis pigmentosa. PloS one. 2017;12(8):e0182389. doi: 10.1371/journal.pone.0182389 28767729 PMC5540409

[18] Ji Y, Yu WQ, Eom YS, Bruce F, Craft CM, Grzywacz NM, et al. The effect of TIMP-1 on the cone mosaic in the retina of the rat model of retinitis pigmentosa. Invest Ophthalmol Vis Sci. 2014;56(1):352–64. doi: 10.1167/iovs.14-15398 25515575 PMC4294290

[19] Shin JA, Eom YS, Yu WQ, Grzywacz NM, Craft CM, Lee EJ. TIMP-1 affects the spatial distribution of dendritic processes of second-order neurons in a rat model of Retinitis Pigmentosa. Experimental eye research. 2015;140:41–52. doi: 10.1016/j.exer.2015.08.005 26277580

[20] Grunwald B, Schoeps B, Kruger A. Recognizing the Molecular Multifunctionality and Interactome of TIMP-1. Trends Cell Biol. 2019;29(1):6–19. doi: 10.1016/j.tcb.2018.08.006 30243515

[21] Ritter LM, Garfield SH, Thorgeirsson UP. Tissue inhibitor of metalloproteinases-1 (TIMP-1) binds to the cell surface and translocates to the nucleus of human MCF-7 breast carcinoma cells. Biochemical and biophysical research communications. 1999;257(2):494–9. doi: 10.1006/bbrc.1999.0408 10198240

[22] Zhao WQ, Li H, Yamashita K, Guo XK, Hoshino T, Yoshida S, et al. Cell cycle-associated accumulation of tissue inhibitor of metalloproteinases-1 (TIMP-1) in the nuclei of human gingival fibroblasts. Journal of cell science. 1998;111 (Pt 9):1147–53. 9547291 10.1242/jcs.111.9.1147

[23] Vichai V, Kirtikara K. Sulforhodamine B colorimetric assay for cytotoxicity screening. Nat Protoc. 2006;1(3):1112–6. doi: 10.1038/nprot.2006.179 17406391

[24] Jeong S, Ledee DR, Gordon GM, Itakura T, Patel N, Martin A, et al. Interaction of clusterin and matrix metalloproteinase-9 and its implication for epithelial homeostasis and inflammation. The American journal of pathology. 2012;180(5):2028–39. doi: 10.1016/j.ajpath.2012.01.025 22440257 PMC3349834

[25] Mosmann T. Rapid colorimetric assay for cellular growth and survival: application to proliferation and cytotoxicity assays. J Immunol Methods. 1983;65(1–2):55–63. doi: 10.1016/0022-1759(83)90303-4 6606682

[26] Jobin PG, Butler GS, Overall CM. New intracellular activities of matrix metalloproteinases shine in the moonlight. Biochimica et biophysica acta Molecular cell research. 2017;1864(11 Pt A):2043–55. doi: 10.1016/j.bbamcr.2017.05.013 28526562

[27] Punzo C, Xiong W, Cepko CL. Loss of daylight vision in retinal degeneration: are oxidative stress and metabolic dysregulation to blame? The Journal of biological chemistry. 2012;287(3):1642–8. doi: 10.1074/jbc.R111.304428 22074929 PMC3265845

[28] Chan CY, Chan YC, Cheuk BL, Cheng SW. Clearance of matrix metalloproteinase-9 is dependent on low-density lipoprotein receptor-related protein-1 expression downregulated by microRNA-205 in human abdominal aortic aneurysm. Journal of vascular surgery. 2017;65(2):509–20. doi: 10.1016/j.jvs.2015.10.065 26781079

[29] Thevenard J, Verzeaux L, Devy J, Etique N, Jeanne A, Schneider C, et al. Low-density lipoprotein receptor-related protein-1 mediates endocytic clearance of tissue inhibitor of metalloproteinases-1 and promotes its cytokine-like activities. PloS one. 2014;9(7):e103839. doi: 10.1371/journal.pone.0103839 25075518 PMC4116228

[30] Jung KK, Liu XW, Chirco R, Fridman R, Kim HR. Identification of CD63 as a tissue inhibitor of metalloproteinase-1 interacting cell surface protein. The EMBO journal. 2006;25(17):3934–42. doi: 10.1038/sj.emboj.7601281 16917503 PMC1560352

[31] Zhang J, Wu T, Zhan S, Qiao N, Zhang X, Zhu Y, et al. TIMP-1 and CD82, a promising combined evaluation marker for PDAC. Oncotarget. 2017;8(4):6496–512. doi: 10.18632/oncotarget.14133 28030805 PMC5351648

[32] Beatty S, Koh H, Phil M, Henson D, Boulton M. The role of oxidative stress in the pathogenesis of age-related macular degeneration. Survey of ophthalmology. 2000;45(2):115–34. doi: 10.1016/s0039-6257(00)00140-5 11033038

[33] Thorgeirsson UP, Yoshiji H, Sinha CC, Gomez DE. Breast cancer; tumor neovasculature and the effect of tissue inhibitor of metalloproteinases-1 (TIMP-1) on angiogenesis. In Vivo. 1996;10(2):137–44. 8744792

[34] Verma G, Datta M. IL-1beta induces ER stress in a JNK dependent manner that determines cell death in human pancreatic epithelial MIA PaCa-2 cells. Apoptosis. 2010;15(7):864–76. doi: 10.1007/s10495-010-0498-4 20411335

[35] Stetler-Stevenson WG. Tissue inhibitors of metalloproteinases in cell signaling: metalloproteinase-independent biological activities. Science signaling. 2008;1(27):re6. doi: 10.1126/scisignal.127re6 18612141 PMC2493614

[36] Roque RS, Imperial CJ, Caldwell RB. Microglial cells invade the outer retina as photoreceptors degenerate in Royal College of Surgeons rats. Invest Ophthalmol Vis Sci. 1996;37(1):196–203. 8550323

[37] Appelbaum T, Santana E, Aguirre GD. Strong upregulation of inflammatory genes accompanies photoreceptor demise in canine models of retinal degeneration. PloS one. 2017;12(5):e0177224. doi: 10.1371/journal.pone.0177224 28486508 PMC5423635

[38] Yoshida N, Ikeda Y, Notomi S, Ishikawa K, Murakami Y, Hisatomi T, et al. Laboratory evidence of sustained chronic inflammatory reaction in retinitis pigmentosa. Ophthalmology. 2013;120(1):e5–12. doi: 10.1016/j.ophtha.2012.07.008 22986110

[39] Dong N, Chang L, Wang B, Chu L. Retinal neuronal MCP-1 induced by AGEs stimulates TNF-alpha expression in rat microglia via p38, ERK, and NF-kappaB pathways. Molecular vision. 2014;20:616–28. 24826069 PMC4016805

[40] Nelson CM, Ackerman KM, O’Hayer P, Bailey TJ, Gorsuch RA, Hyde DR. Tumor necrosis factor-alpha is produced by dying retinal neurons and is required for Muller glia proliferation during zebrafish retinal regeneration. The Journal of neuroscience: the official journal of the Society for Neuroscience. 2013;33(15):6524–39. 10.1523/JNEUROSCI.3838-12.2013 PMC3740543 23575850

[41] Nagase H, Visse R, Murphy G. Structure and function of matrix metalloproteinases and TIMPs. Cardiovasc Res. 2006;69(3):562–73. doi: 10.1016/j.cardiores.2005.12.002 16405877

[42] Kohrmann A, Kammerer U, Kapp M, Dietl J, Anacker J. Expression of matrix metalloproteinases (MMPs) in primary human breast cancer and breast cancer cell lines: New findings and review of the literature. BMC cancer. 2009;9:188. doi: 10.1186/1471-2407-9-188 19531263 PMC2706257

[43] Hill JW, Poddar R, Thompson JF, Rosenberg GA, Yang Y. Intranuclear matrix metalloproteinases promote DNA damage and apoptosis induced by oxygen-glucose deprivation in neurons. Neuroscience. 2012;220:277–90. doi: 10.1016/j.neuroscience.2012.06.019 22710064 PMC4546359

[44] Yang Y, Candelario-Jalil E, Thompson JF, Cuadrado E, Estrada EY, Rosell A, et al. Increased intranuclear matrix metalloproteinase activity in neurons interferes with oxidative DNA repair in focal cerebral ischemia. Journal of neurochemistry. 2010;112(1):134–49. doi: 10.1111/j.1471-4159.2009.06433.x 19840223 PMC4950937

[45] Sbai O, Ould-Yahoui A, Ferhat L, Gueye Y, Bernard A, Charrat E, et al. Differential vesicular distribution and trafficking of MMP-2, MMP-9, and their inhibitors in astrocytes. Glia. 2010;58(3):344–66. doi: 10.1002/glia.20927 19780201

[46] Sinha SK, Asotra K, Uzui H, Nagwani S, Mishra V, Rajavashisth TB. Nuclear localization of catalytically active MMP-2 in endothelial cells and neurons. American journal of translational research. 2014;6(2):155–62. 24489995 PMC3902226

[47] Si-Tayeb K, Monvoisin A, Mazzocco C, Lepreux S, Decossas M, Cubel G, et al. Matrix metalloproteinase 3 is present in the cell nucleus and is involved in apoptosis. The American journal of pathology. 2006;169(4):1390–401. doi: 10.2353/ajpath.2006.060005 17003494 PMC1780186

[48] Zimowska M, Swierczynska M, Ciemerych MA. Nuclear MMP-9 role in the regulation of rat skeletal myoblasts proliferation. Biol Cell. 2013;105(8):334–44. doi: 10.1111/boc.201300020 23646930

[49] Marchant DJ, Bellac CL, Moraes TJ, Wadsworth SJ, Dufour A, Butler GS, et al. A new transcriptional role for matrix metalloproteinase-12 in antiviral immunity. Nat Med. 2014;20(5):493–502. doi: 10.1038/nm.3508 24784232

[50] Cuadrado E, Rosell A, Borrell-Pages M, Garcia-Bonilla L, Hernandez-Guillamon M, Ortega-Aznar A, et al. Matrix metalloproteinase-13 is activated and is found in the nucleus of neural cells after cerebral ischemia. Journal of cerebral blood flow and metabolism: official journal of the International Society of Cerebral Blood Flow and Metabolism. 2009;29(2):398–410. 10.1038/jcbfm.2008.130 18985055

[51] Ip YC, Cheung ST, Fan ST. Atypical localization of membrane type 1-matrix metalloproteinase in the nucleus is associated with aggressive features of hepatocellular carcinoma. Mol Carcinog. 2007;46(3):225–30. doi: 10.1002/mc.20270 17219425

[52] Zuo X, Pan W, Feng T, Shi X, Dai J. Matrix metalloproteinase 3 promotes cellular anti-dengue virus response via interaction with transcription factor NFkappaB in cell nucleus. PloS one. 2014;9(1):e84748. doi: 10.1371/journal.pone.0084748 24416274 PMC3885614

[53] Xie Y, Lu W, Liu S, Yang Q, Goodwin JS, Sathyanarayana SA, et al. MMP7 interacts with ARF in nucleus to potentiate tumor microenvironments for prostate cancer progression in vivo. Oncotarget. 2016;7(30):47609–19. doi: 10.18632/oncotarget.10251 27356744 PMC5216965

[54] Xie Y, Mustafa A, Yerzhan A, Merzhakupova D, Yerlan P, A NO, et al. Nuclear matrix metalloproteinases: functions resemble the evolution from the intracellular to the extracellular compartment. Cell Death Discov. 2017;3:17036. doi: 10.1038/cddiscovery.2017.36 28811933 PMC5554797

[55] Hahn-Dantona E, Ruiz JF, Bornstein P, Strickland DK. The low density lipoprotein receptor-related protein modulates levels of matrix metalloproteinase 9 (MMP-9) by mediating its cellular catabolism. The Journal of biological chemistry. 2001;276(18):15498–503. doi: 10.1074/jbc.M100121200 11279011

[56] Zurhove K, Nakajima C, Herz J, Bock HH, May P. Gamma-secretase limits the inflammatory response through the processing of LRP1. Science signaling. 2008;1(47):ra15. doi: 10.1126/scisignal.1164263 19036715 PMC2694618

[57] Li H, Nishio K, Yamashita K, Hayakawa T, Hoshino T. Cell cycle-dependent localization of tissue inhibitor of metalloproteinases-1 immunoreactivity in cultured human gingival fibroblasts. Nagoya journal of medical science. 1995;58(3–4):133–42. 8725497

[58] Bourgeois B, Hutten S, Gottschalk B, Hofweber M, Richter G, Sternat J, et al. Nonclassical nuclear localization signals mediate nuclear import of CIRBP. Proceedings of the National Academy of Sciences of the United States of America. 2020;117(15):8503–14. doi: 10.1073/pnas.1918944117 32234784 PMC7165476

[59] Spector DL, Lamond AI. Nuclear speckles. Cold Spring Harbor perspectives in biology. 2011;3(2). doi: 10.1101/cshperspect.a000646 20926517 PMC3039535

[60] An H, Tan JT, Shelkovnikova TA. Stress granules regulate stress-induced paraspeckle assembly. The Journal of cell biology. 2019;218(12):4127–40. doi: 10.1083/jcb.201904098 31636118 PMC6891081

[61] Bhatt L, Groeger G, McDermott K, Cotter TG. Rod and cone photoreceptor cells produce ROS in response to stress in a live retinal explant system. Molecular vision. 2010;16:283–93. 20177432 PMC2825485

[62] Groeger G, Mackey AM, Pettigrew CA, Bhatt L, Cotter TG. Stress-induced activation of Nox contributes to cell survival signalling via production of hydrogen peroxide. Journal of neurochemistry. 2009;109(5):1544–54. doi: 10.1111/j.1471-4159.2009.06081.x 19344371

[63] Mackey AM, Sanvicens N, Groeger G, Doonan F, Wallace D, Cotter TG. Redox survival signalling in retina-derived 661W cells. Cell Death Differ. 2008;15(8):1291–303. doi: 10.1038/cdd.2008.43 18404155

[64] Bellezza I. Oxidative Stress in Age-Related Macular Degeneration: Nrf2 as Therapeutic Target. Frontiers in pharmacology. 2018;9:1280. doi: 10.3389/fphar.2018.01280 30455645 PMC6230566

[65] Rhee SG. Cell signaling. H2O2, a necessary evil for cell signaling. Science. 2006;312(5782):1882–3. doi: 10.1126/science.1130481 16809515

[66] Gough DR, Cotter TG. Hydrogen peroxide: a Jekyll and Hyde signalling molecule. Cell death & disease. 2011;2:e213. doi: 10.1038/cddis.2011.96 21975295 PMC3219092

[67] Wang J, Shanmugam A, Markand S, Zorrilla E, Ganapathy V, Smith SB. Sigma 1 receptor regulates the oxidative stress response in primary retinal Muller glial cells via NRF2 signaling and system xc(-), the Na(+)-independent glutamate-cystine exchanger. Free radical biology & medicine. 2015;86:25–36. doi: 10.1016/j.freeradbiomed.2015.04.009 25920363 PMC4554890

[68] Agca C, Boldt K, Gubler A, Meneau I, Corpet A, Samardzija M, et al. Expression of leukemia inhibitory factor in Muller glia cells is regulated by a redox-dependent mRNA stability mechanism. BMC Biol. 2015;13:30. doi: 10.1186/s12915-015-0137-1 25907681 PMC4462110

[69] Kanda A, Noda K, Hirose I, Ishida S. TGF-beta-SNAIL axis induces Muller glial-mesenchymal transition in the pathogenesis of idiopathic epiretinal membrane. Scientific reports. 2019;9(1):673. doi: 10.1038/s41598-018-36917-9 30679596 PMC6346093

[70] Lee SY, Ju MK, Jeon HM, Lee YJ, Kim CH, Park HG, et al. Reactive oxygen species induce epithelialmesenchymal transition, glycolytic switch, and mitochondrial repression through the Dlx2/Snail signaling pathways in MCF7 cells. Molecular medicine reports. 2019;20(3):2339–46. doi: 10.3892/mmr.2019.10466 31322179

[71] Alvarez M, Estivill X, de la Luna S. DYRK1A accumulates in splicing speckles through a novel targeting signal and induces speckle disassembly. Journal of cell science. 2003;116(Pt 15):3099–107. doi: 10.1242/jcs.00618 12799418

[72] Yao C, Jasmer DP. Trichinella spiralis-infected muscle cells: abundant RNA polymerase II in nuclear speckle domains colocalizes with nuclear antigens. Infect Immun. 2001;69(6):4065–71. doi: 10.1128/IAI.69.6.4065-4071.2001 11349077 PMC98470

[73] Roesch K, Stadler MB, Cepko CL. Gene expression changes within Muller glial cells in retinitis pigmentosa. Molecular vision. 2012;18:1197–214. 22665967 PMC3365136

[74] Tuuttila A, Morgunova E, Bergmann U, Lindqvist Y, Maskos K, Fernandez-Catalan C, et al. Three-dimensional structure of human tissue inhibitor of metalloproteinases-2 at 2.1 A resolution. Journal of molecular biology. 1998;284(4):1133–40. doi: 10.1006/jmbi.1998.2223 9837731

[75] Williamson RA, Martorell G, Carr MD, Murphy G, Docherty AJ, Freedman RB, et al. Solution structure of the active domain of tissue inhibitor of metalloproteinases-2. A new member of the OB fold protein family. Biochemistry. 1994;33(39):11745–59. doi: 10.1021/bi00205a010 7918391

[76] Flynn RL, Zou L. Oligonucleotide/oligosaccharide-binding fold proteins: a growing family of genome guardians. Critical reviews in biochemistry and molecular biology. 2010;45(4):266–75. doi: 10.3109/10409238.2010.488216 20515430 PMC2906097

[77] Srinivas US, Tan BWQ, Vellayappan BA, Jeyasekharan AD. ROS and the DNA damage response in cancer. Redox Biol. 2019;25:101084. doi: 10.1016/j.redox.2018.101084 30612957 PMC6859528

[78] Davalli P, Marverti G, Lauriola A, D’Arca D. Targeting Oxidatively Induced DNA Damage Response in Cancer: Opportunities for Novel Cancer Therapies. Oxid Med Cell Longev. 2018;2018:2389523. doi: 10.1155/2018/2389523 29770165 PMC5892224

[79] Wang YH, Hariharan A, Bastianello G, Toyama Y, Shivashankar GV, Foiani M, et al. DNA damage causes rapid accumulation of phosphoinositides for ATR signaling. Nat Commun. 2017;8(1):2118. doi: 10.1038/s41467-017-01805-9 29242514 PMC5730617

