# Supplementary figures and images for: Matrix metalloproteinase-9 (MMP-9) and tissue inhibitor of metalloproteinases 1 (TIMP-1) are localized in the nucleus of retinal Müller glial cells and modulated by cytokines and oxidative stress
Source: PLoS One. 2021 Jul 16;16(7):e0253915. doi: 10.1371/journal.pone.0253915 (PMC8284794; doi:10.1371/journal.pone.0253915)

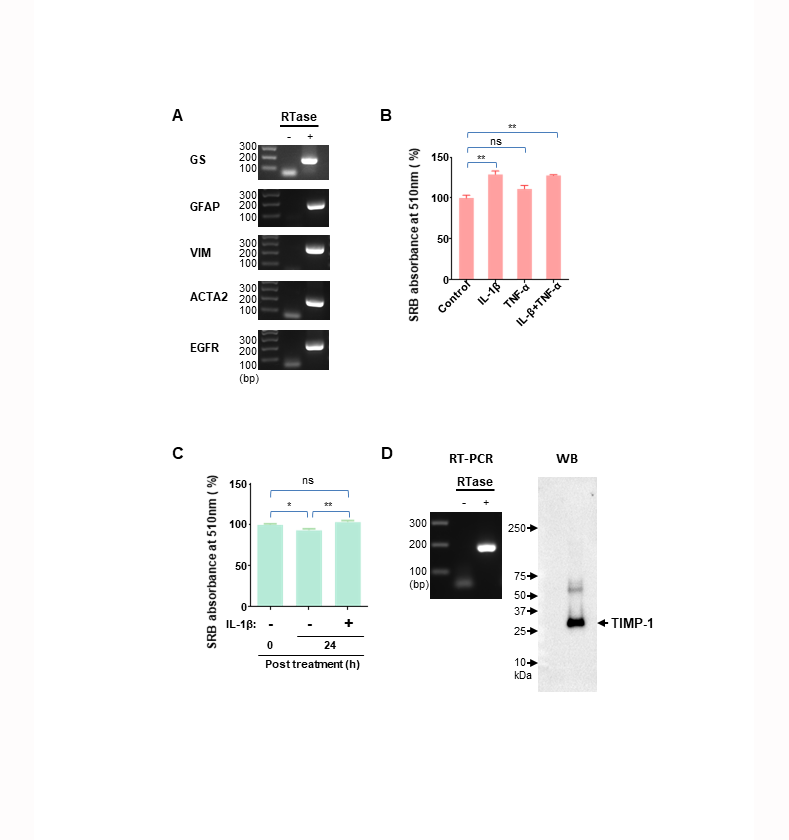

Supplement: S1 Fig — (A) Total RNA was isolated from MIO-M1 cells to synthesize cDNA, which was used for transcript-specific RT-PCR for Müller glial cell marker proteins. Reverse transcriptase-omitted samples (-RTase) were included as a negative control. GS, glutamine sythetase; GFAP, glial fibrillary acidic protein; VIM, vimentin; ACTA2, α-smooth muscle actin; EGFR, epidermal growth factor receptor. (B) MIO-M1 cells were treated or untreated with IL-β and TNF-α, at 10 ng/mL, alone or in combination, for 24 h, and then subjected to SRB assay to measure viable cell densities. Control refers to untreated samples. Relative cell densities are presented as % mean ± SE (n = 3), with Control set as 100%. IL-1β increases the SRB absorbance to 129% (p<0.01 vs. Control), and TNF-α, to 111% (p>0.05 vs. Control). The combination treatment also increases the growth rate by 128% (p<0.01 vs. Control); **, p<0.01 (vs. Control). (C) MIO-M1 cells were cultured in FBS-free media for 0 h and 24 h with and without 10 ng/mL IL-1β treatment, followed by SRB assay (n = 6). The cell density was reduced to 94% (p<0.05) at 24h from 100% at 0h without IL-1β treatment; however, the density was preserved at 104% (p<0.01) at 24 h with the treatment. *, p<0.05; **, p<0.01; ns, not significant (p>0.05). (D) The expression of TIMP-1 from MIO-M1 cells was confirmed. (Left) transcript-specific RT-PCR using cDNA. (Right) Immunoblot analysis (WB) was done using the conditioned media with anti-TIMP-1 antibody (Cell Signaling Tech, Catalog number:8946), TIMP-1 protein band is indicated by an arrow. (TIF) [file pone.0253915.s001.tif]

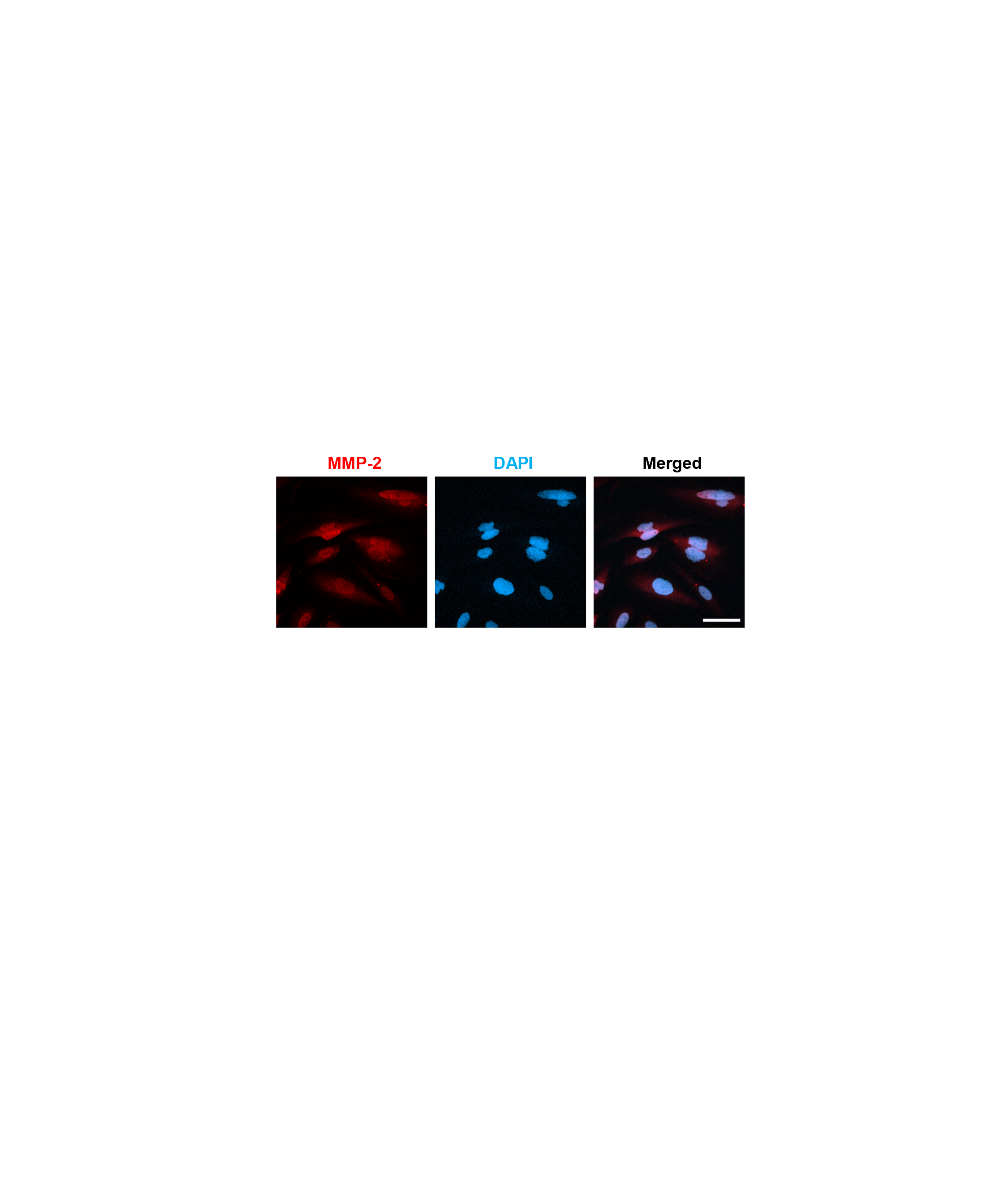

Supplement: S2 Fig — MIO-M1 cells cultured in regular media were subjected to IHC fluorescent confocal microscopy. The cells were immunohistologically stained with MMP-2 antibody (red) to localize the proteins. Nuclear region is defined by chromatin staining with DAPI (blue). Scale bar, 50 μm. (TIF) [file pone.0253915.s002.tif]

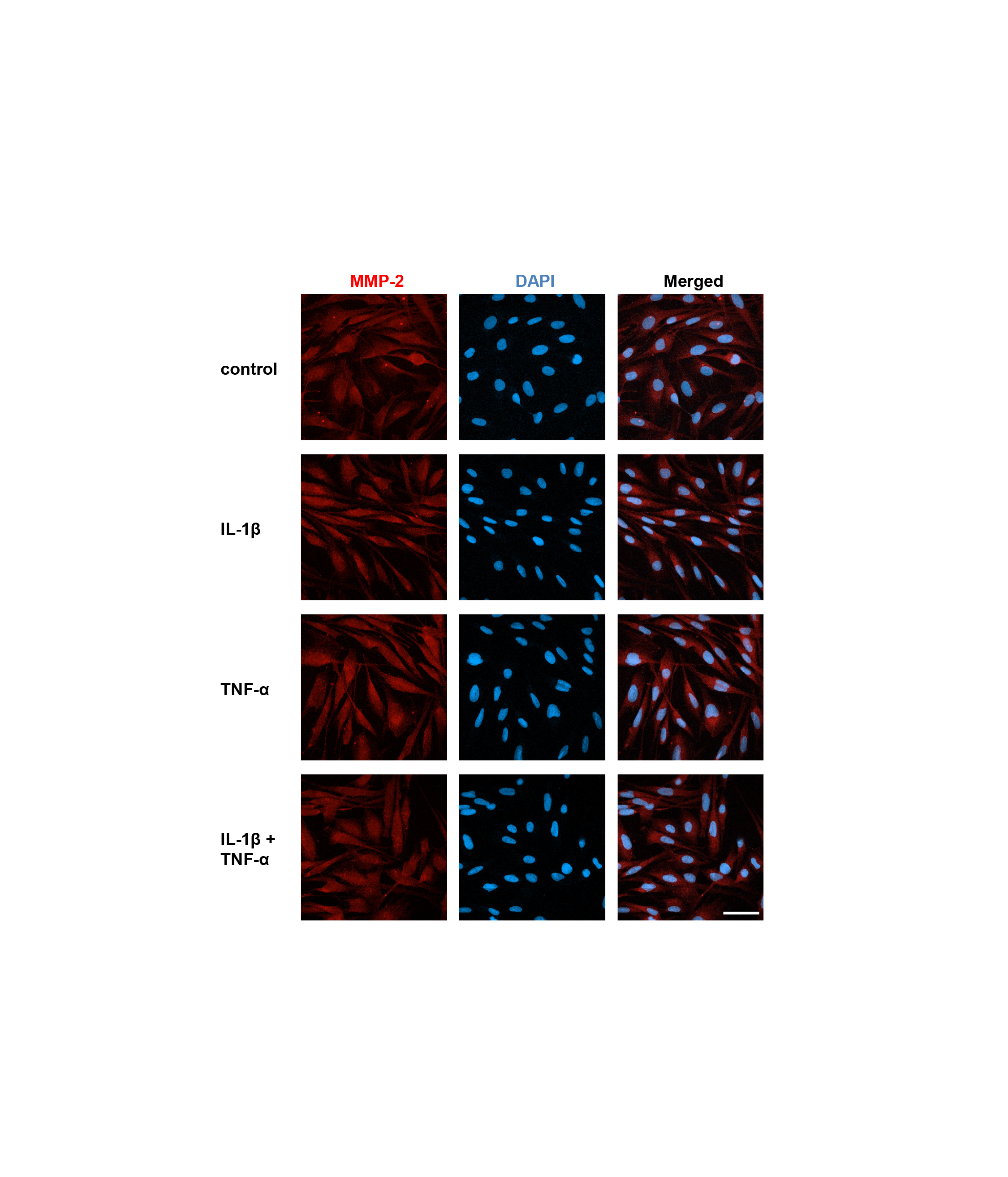

Supplement: S3 Fig — MIO-M1 cells were treated or untreated with IL-1β and/or TNF-α, each at 10 ng/mL in FBS-free media, alone or in combination, for 24 h, and then IHC was performed to detect intracellular MMP-2 (red). Nuclear region is defined by chromatin staining with DAPI (blue). Representative micrograms of each treatment group are presented. Scale bar, 50 μm. (TIF) [file pone.0253915.s003.tif]

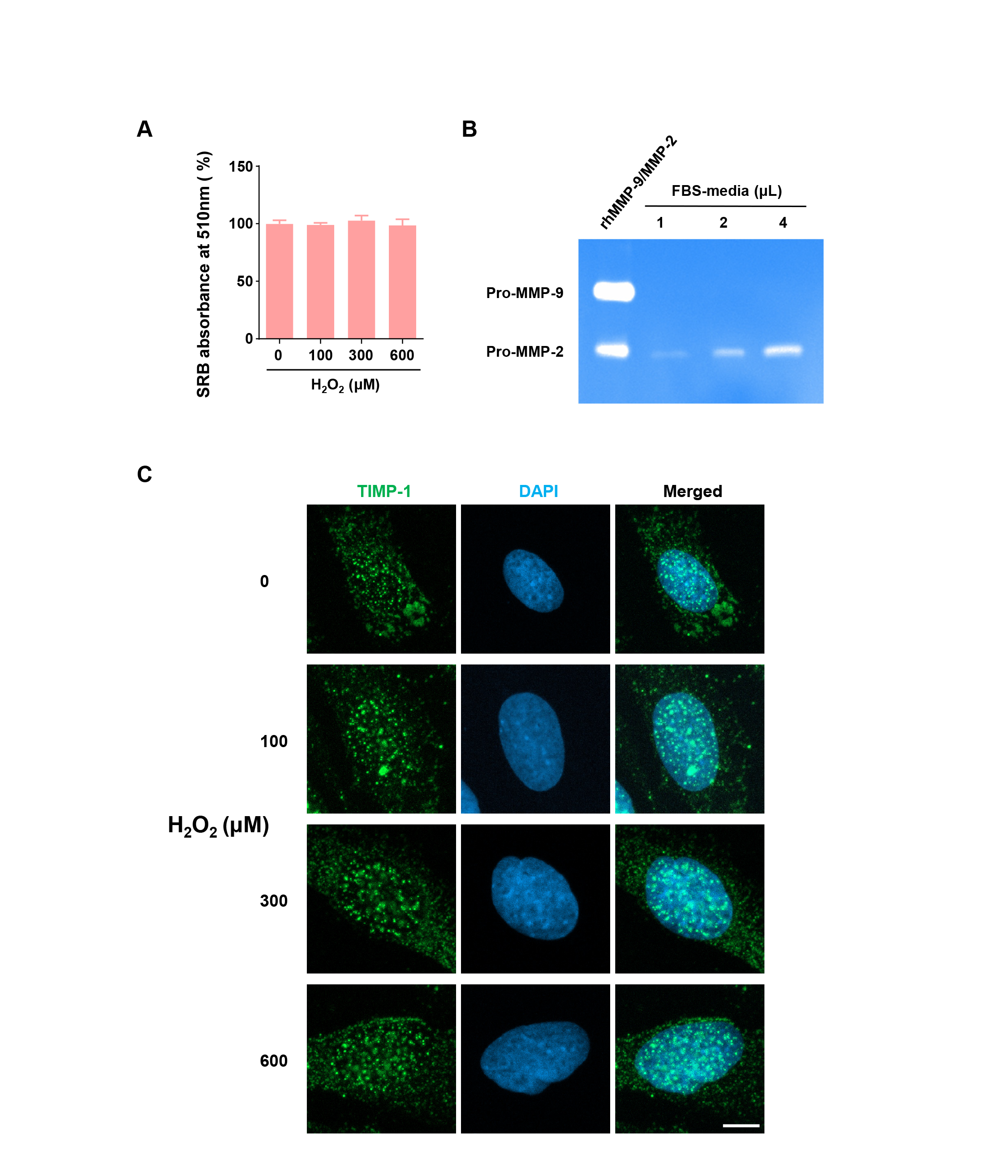

Supplement: S4 Fig — (A) MIO-M1 cells were treated with H2O2 at 0μM, 100μM, 300μM, and 600μM in FBS-free media, for 24 h, and then subjected to SRB assay to measure cell densities. Relative cell densities are presented as % mean ± SE (n = 3), with control (0μM) set as 100%. There are no significant changes among treatment groups in SRB absorbance value. (B) Standard culture media containing 10% FBS were subjected to gelatin zymography to show that bovine serum contains MMP-2, which has a molecular size similar to human MMP-2. (C) MIO-M1 cells were cultured in the presence of H2O2, at 0μM and 100μM, in FBS-free media, for 24 h, and then subjected to IHC for TIMP-1 (green) and DAPI (blue) micrograms. Single cell micrograms are presented. Scale bar, 10 μm. (TIF) [file pone.0253915.s004.tif]
