# Supplementary material for: Matrix metalloproteinase-9 (MMP-9) and tissue inhibitor of metalloproteinases 1 (TIMP-1) are localized in the nucleus of retinal Müller glial cells and modulated by cytokines and oxidative stress
Source: PLoS One. 2021 Jul 16;16(7):e0253915. doi: 10.1371/journal.pone.0253915 (PMC8284794; doi:10.1371/journal.pone.0253915)
Supplement: S1 Table — (DOCX) [file pone.0253915.s005.docx]

| **Target** | **Primary Antibody Name** | **Manufacturer** | **Catalog Number** | **Dilution Rate** |
| --- | --- | --- | --- | --- |
| β Actin | Anti-β−Actin [AC-15] | Abcam | Ab6276 | 1:1000 |
| CD63 | Anti-CD63 clone RFAC4 | Millipore | CBL553 | 1:500 |
| CD82 | Anti-CD82 [C33] | Abcam | Ab140238 | 1:500 |
| LRP1 | Anti-LRP1 Recombinant [EPR3724] | Abcam | Ab92544 | 1:500 |
| MMP-2 | Anti-MMP-2, amino acid 468-483 hMMP2, clone 42-5D11 | Millipore | MAB3308 | 1:500 |
| MMP-9 | Anti-MMP9 [56-2A4] | Abcam | Ab58803 | 1:500 |
| TIMP-1 | Anti-TIMP-1 | Millipore | Ab770 | 1:500 |
|  | **Secondary Antibody** | **Manufacturer** | **Catalog Number** | **Dilution Rate** |
| Rabbit IgG | Donkey anti-Rabbit IgG (H+L) Highly Cross-Adsorbed, Alexa Fluor 488 | Invitrogen | A21206 | 1:500 |
| Mouse IgG | Goat anti-Mouse IgG (H+L) Highly Cross-Adsorbed, Alexa Fluor 488 | Invitrogen | A11029 | 1:500 |
| Mouse IgG | Donkey anti-Mouse IgG (H+L) Highly Cross-Adsorbed, Alexa Fluor 568 | Invitrogen | A10037 | 1:500 |
| Rabbit IgG | Goat anti-Rabbit IgG (H+L) Highly Cross-Adsorbed, Alexa Fluor 568 | Invitrogen | A11036 | 1:500 |
