# Supplementary material for: Matrix metalloproteinase-9 (MMP-9) and tissue inhibitor of metalloproteinases 1 (TIMP-1) are localized in the nucleus of retinal Müller glial cells and modulated by cytokines and oxidative stress
Source: PLoS One. 2021 Jul 16;16(7):e0253915. doi: 10.1371/journal.pone.0253915 (PMC8284794; doi:10.1371/journal.pone.0253915)
Supplement: S2 Table — (DOCX) [file pone.0253915.s006.docx]

| Genes | Primer DNA sequences | DNA sizes (bp) |
| --- | --- | --- |
| TIMP-1 | F: 5'-CTGCAATTCCGACCTCGTC-3'  R: 5'-GGAAGTATCCGCAGACACTCTC-3' | 185 |
| CD63 | F: 5'-GACAGGATGCAGGCAGATT-3'  R: 5'-GCAGCTACCACCAGCACA-3' | 215 |
| CD82 | F: 5'-GGGGGAAGAGGACAACAGC-3'  R: 5'-CCATCCCCAGGAGCTCG-3' | 203 |
| LRP1 | F: 5'-CGAGGCCCCTGAGATTTGT-3'  R: 5'-CATCGAGTGTGGGGACAC-3' | 215 |
| GS | F: 5'-AATCGAAGGCCTGCAGAGAC-3'  R: 5'-CACCACAGTAATATGGACCCTG-3' | 181 |
| GFAP | F: 5'-GGCTGCCTATAGACAGGAAGC-3'  R: 5'-GCTTGGCCACGTCAAGC-3' | 179 |
| VIM | F: 5'-GTCCCTCACCTGTGAAGTGG-3'  R: 5'-AAGAGGCAGAGAAATCCTGCTC-3' | 273 |
| ACTA2 | F: 5'-GACGACATGGAAAAGATCTGG-3'  R: 5'-CCAGAGGCATAGAGAGACAGCAC-3' | 200 |
| EGFR | F: 5'-ATGTCGATGGACTTCCAGAACC-3'  R: 5'-GAATTTGCGGCAGACCAG-3' | 237 |
