# Supplementary material for: Matrix metalloproteinase-9 (MMP-9) and tissue inhibitor of metalloproteinases 1 (TIMP-1) are localized in the nucleus of retinal Müller glial cells and modulated by cytokines and oxidative stress
Source: PLoS One. 2021 Jul 16;16(7):e0253915. doi: 10.1371/journal.pone.0253915 (PMC8284794; doi:10.1371/journal.pone.0253915)
Supplement: S1 Raw images — (PDF) [file pone.0253915.s007.pdf]

Fig 1 B

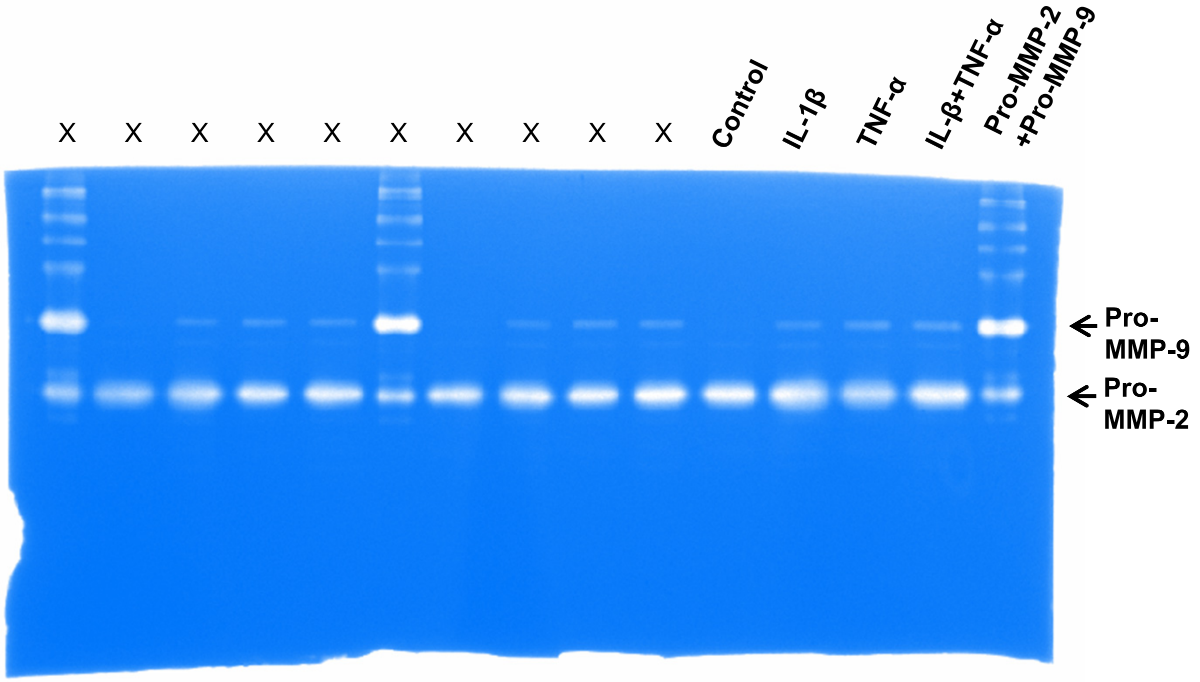

Method: SDS-PAGE gels were pre-stained with Coomassie Blue Stain were imaged by Bio-Rad Gel Doc XR+ Gel Documentation System. Images were captured by Bio-Rad Image Lab image acquisition and analysis software.

Fig 4 B

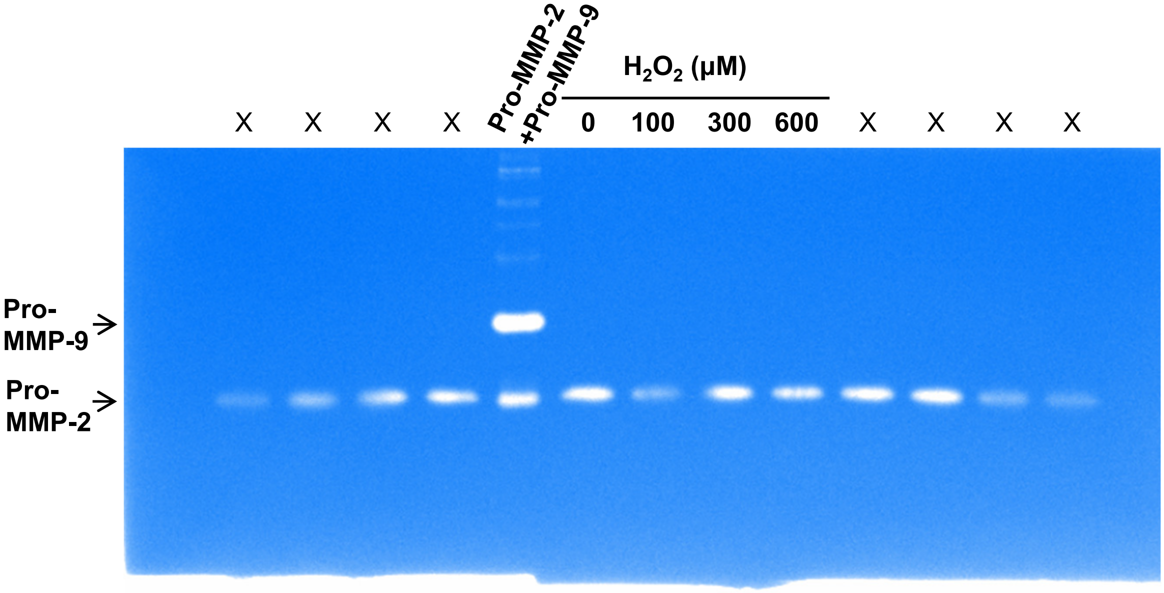

Method: SDS-PAGE gels were pre-stained with Coomassie Blue Stain were imaged by Bio-Rad Gel Doc XR+ Gel Documentation System. Images were captured by Bio-Rad Image Lab image acquisition and analysis software.

Fig 5 A

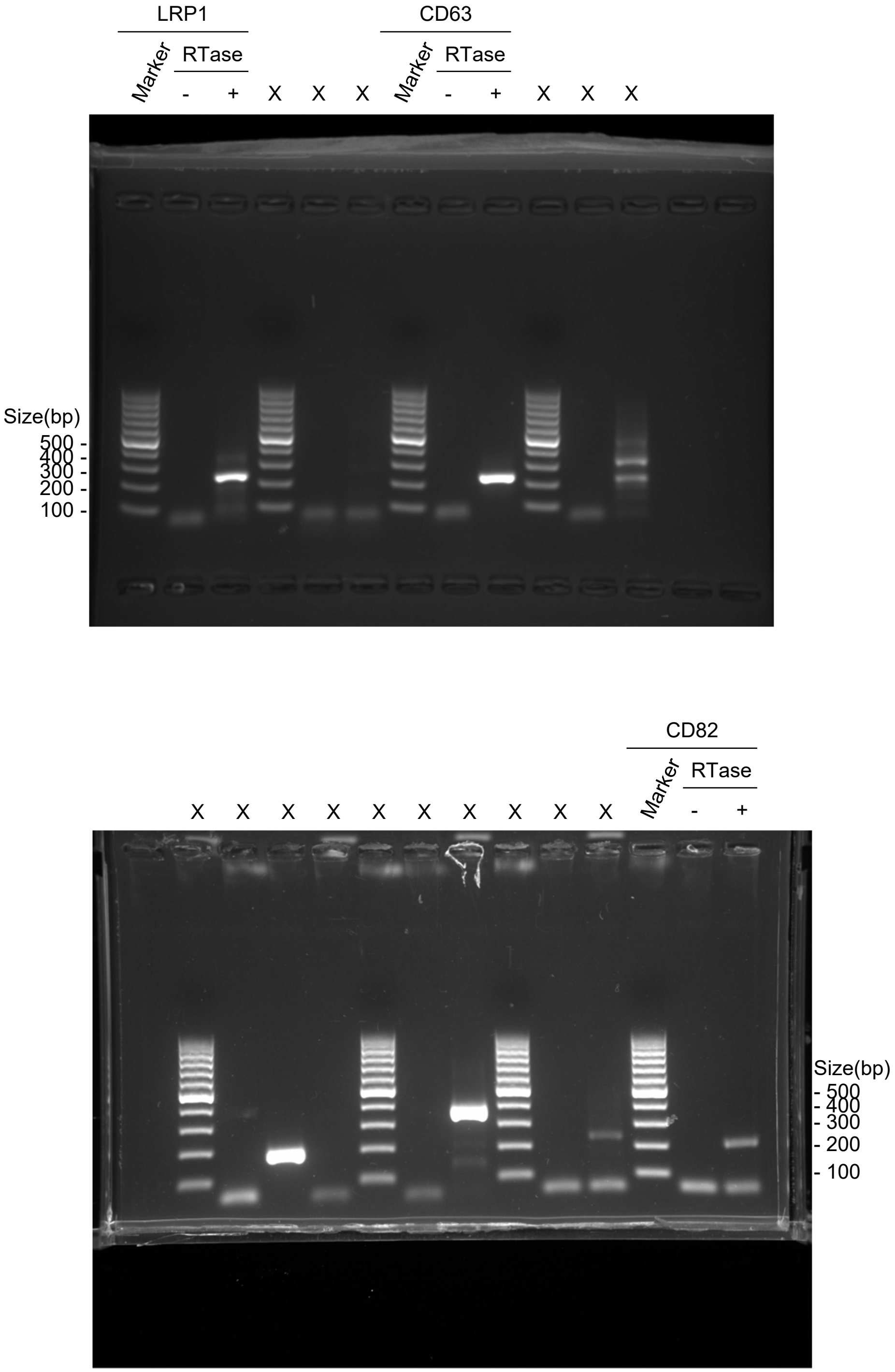

Method: Agarose gels pre-stained with GelRed® Nucleic Acid Gel Stain were imaged by Bio-Rad Gel Doc XR+ Gel Documentation System. Images were captured by Bio-Rad Image Lab image acquisition and analysis software.

Supplemental Fig 1 A

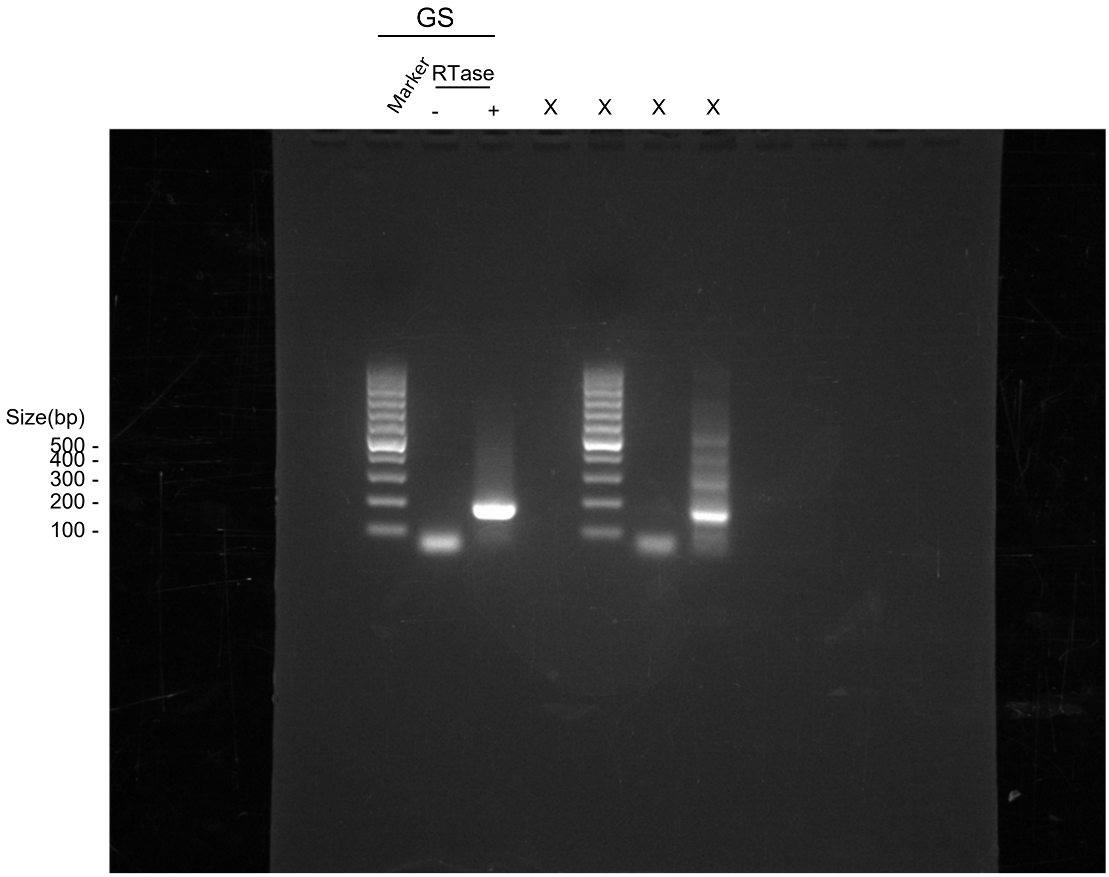

Method: Agarose gels pre-stained with GelRed® Nucleic Acid Gel Stain were imaged by Bio-Rad Gel Doc XR+ Gel Documentation System. Images were captured by Bio-Rad Image Lab image acquisition and analysis software.

Supplemental Fig 1 A

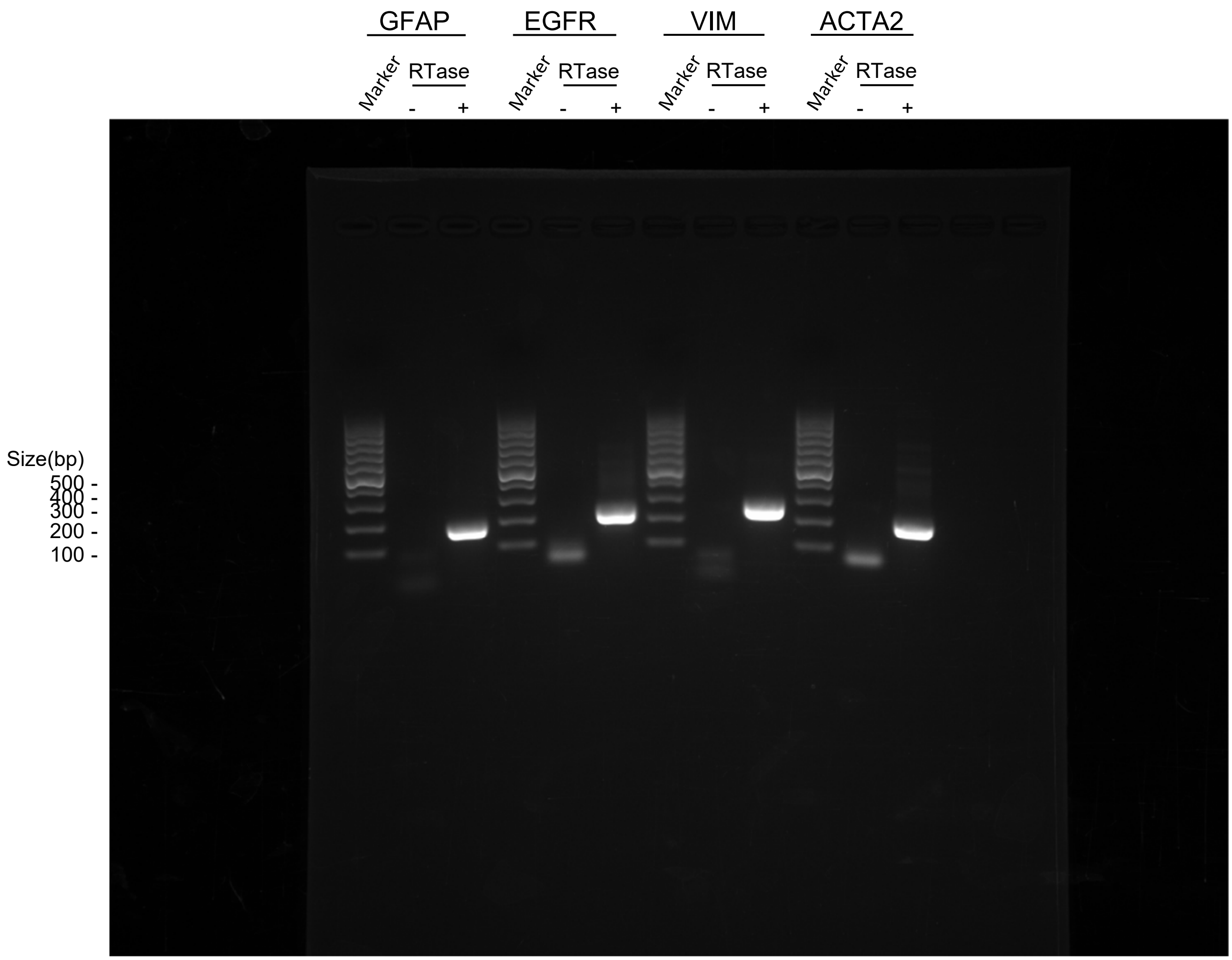

Method: Agarose gels pre-stained with GelRed® Nucleic Acid Gel Stain were imaged by Bio-Rad Gel Doc XR+ Gel Documentation System. Images were captured by Bio-Rad Image Lab image acquisition and analysis software.

Supplemental Fig 1 D

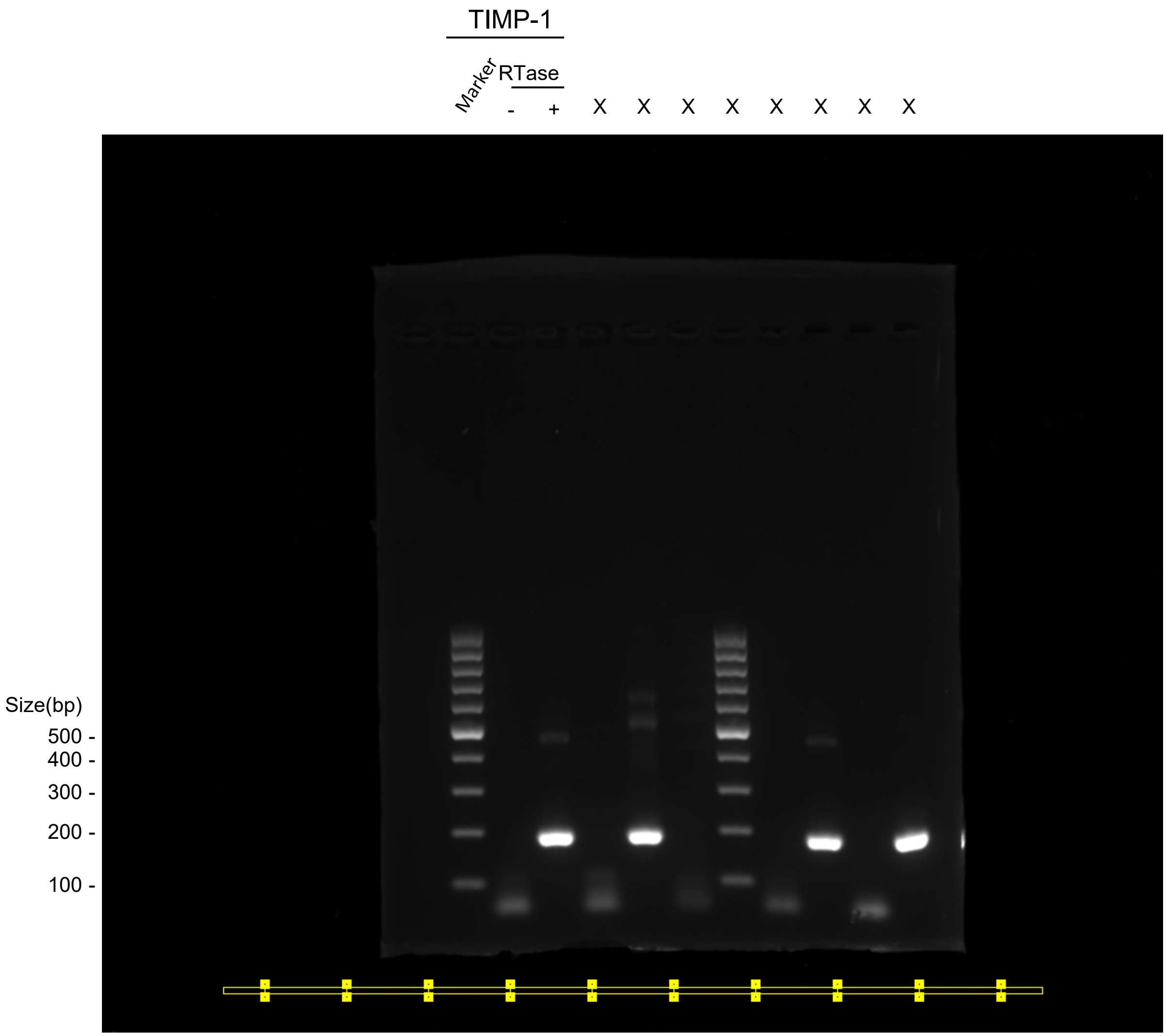

Method: Agarose gels pre-stained with GelRed® Nucleic Acid Gel Stain were imaged by Bio-Rad Gel Doc XR+ Gel Documentation System. Images were captured by Bio-Rad Image Lab image acquisition and analysis software.

Supplemental Fig 1 D

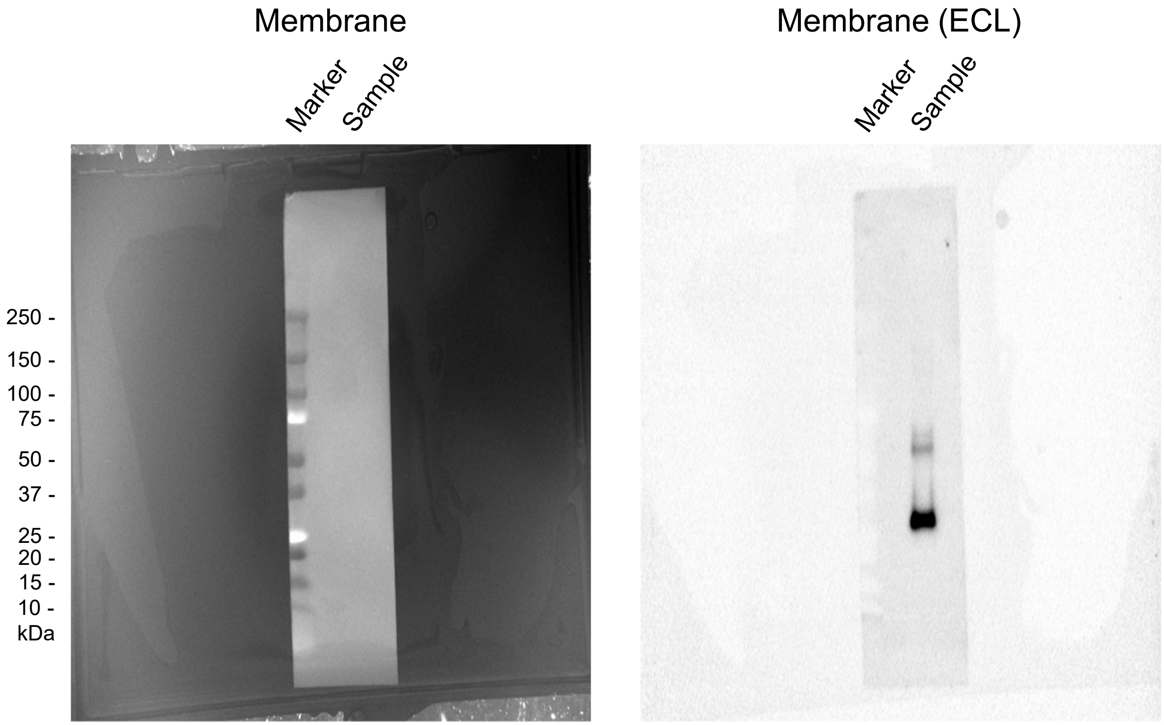

Method: PVDF membrane stained with anti-TIMP-1 antibody were immersed with ECL solution, then imaged by Bio-Rad Gel Doc XR+ Gel Documentation System. Images were captured by Bio-Rad Image Lab image acquisition and analysis software.

Supplemental Fig 4 B

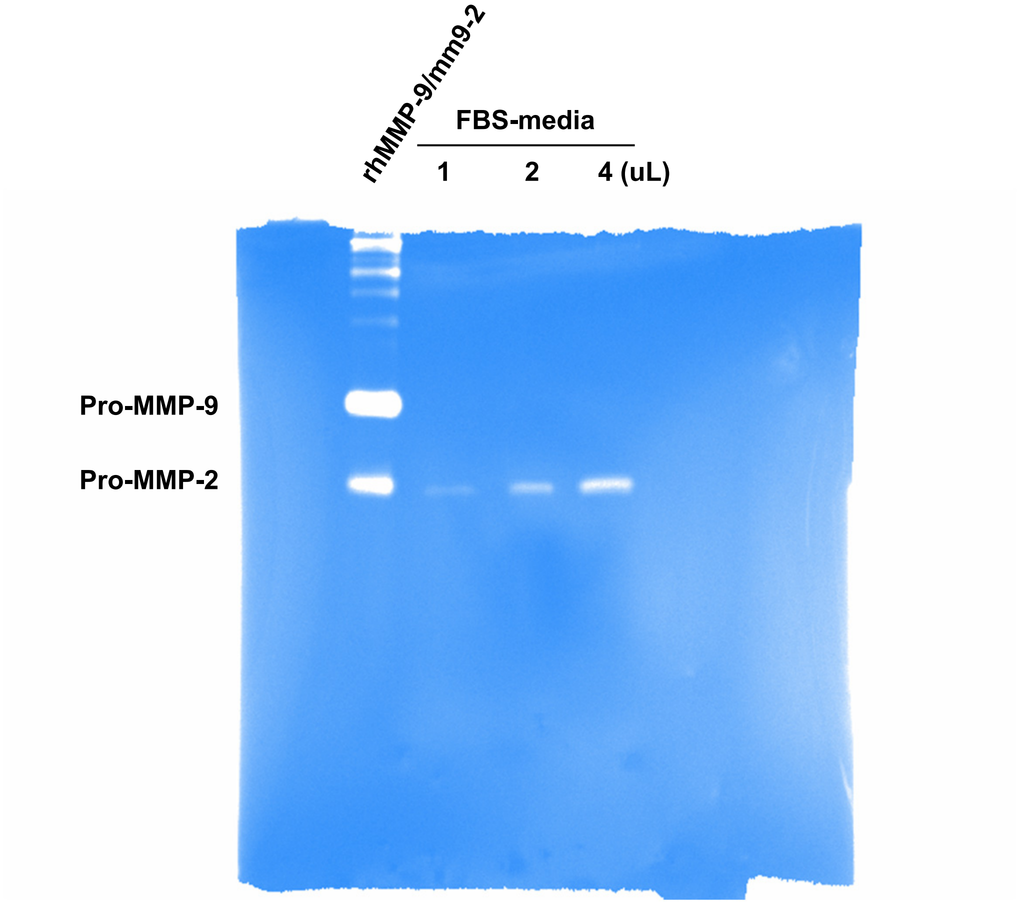

Method: SDS-PAGE gels were pre-stained with Coomassie Blue Stain were imaged by Bio-Rad Gel Doc XR+ Gel Documentation System. Images were captured by Bio-Rad Image Lab image acquisition and analysis software.
